# DeepConv-DTI: Prediction of drug-target interactions via deep learning with convolution on protein sequences

**DOI:** 10.1371/journal.pcbi.1007129

**Published:** 2019-06-14

**Authors:** Ingoo Lee, Jongsoo Keum, Hojung Nam

**Affiliations:** School of Electrical Engineering and Computer Science, Gwangju Institute of Science and Technology, Buk-ku, Gwangju, Republic of Korea; University of Houston, UNITED STATES

## Abstract

Identification of drug-target interactions (DTIs) plays a key role in drug discovery. The high cost and labor-intensive nature of *in vitro* and *in vivo* experiments have highlighted the importance of *in silico*-based DTI prediction approaches. In several computational models, conventional protein descriptors have been shown to not be sufficiently informative to predict accurate DTIs. Thus, in this study, we propose a deep learning based DTI prediction model capturing local residue patterns of proteins participating in DTIs. When we employ a convolutional neural network (CNN) on raw protein sequences, we perform convolution on various lengths of amino acids subsequences to capture local residue patterns of generalized protein classes. We train our model with large-scale DTI information and demonstrate the performance of the proposed model using an independent dataset that is not seen during the training phase. As a result, our model performs better than previous protein descriptor-based models. Also, our model performs better than the recently developed deep learning models for massive prediction of DTIs. By examining pooled convolution results, we confirmed that our model can detect binding sites of proteins for DTIs. In conclusion, our prediction model for detecting local residue patterns of target proteins successfully enriches the protein features of a raw protein sequence, yielding better prediction results than previous approaches. Our code is available at https://github.com/GIST-CSBL/DeepConv-DTI.

## Author summary

Drugs work by interacting with target proteins to activate or inhibit a target’s biological process. Therefore, identification of DTIs is a crucial step in drug discovery. However, identifying drug candidates via biological assays is very time and cost consuming, which introduces the need for a computational prediction approach for the identification of DTIs. In this work, we constructed a novel DTI prediction model to extract local residue patterns of target protein sequences using a CNN-based deep learning approach. As a result, the detected local features of protein sequences perform better than other protein descriptors for DTI prediction and previous models for predicting PubChem independent test datasets. That is, our approach of capturing local residue patterns with CNN successfully enriches protein features from a raw sequence.

## Introduction

The identification of drug-target interactions (DTIs) plays a key role in the early stage of drug discovery. Thus, drug developers screen for compounds that interact with specified targets with biological activities of interest. However, the identification of DTIs in large-scale chemical or biological experiments usually takes 2~3 years of experiments, with high associated costs [1]. Therefore, with the accumulation of drugs, targets, and interaction data, various computational methods have been developed for the prediction of possible DTIs to aid in drug discovery.

Among computational approaches, docking methods, which simulate the binding of a small molecule and a protein using 3D structure, were initially studied. Docking methods recruit various scoring functions and mode definitions to minimize free energy for binding. Docking methods have advanced by themselves, and recently, the Docking Approach using Ray-Casting (DARC) model identified 21 compounds by using an elaborate binding pocket topography mapping methodology, and the results were reproduced in a biochemical assay [2]. In addition, studies have examined several similarity-based methods in which it was assumed that drugs bind to proteins similar to known targets and vice versa. One of the early methods is that of *Yamanashi et al*., which utilized a kernel regression method to use the information on known drug interactions as the input to identify new DTIs, combining a chemical space and genomic spaces into a pharmacological space [3]. To overcome the requirement of the bipartite model for massive computational power, *Beakley et al*. developed the bipartite local model, which trains the interaction model locally but not globally. In addition to substantially reducing the computational complexity, this model exhibited higher performance than the previous model [4]. As another approach to DTI prediction models, matrix factorization methods have been recruited to predict DTIs, which approximate multiplying two latent matrices representing the compound and target protein to an interaction matrix and similarity score matrix [5, 6]. In this work, regularized matrix factorization methods successfully learn the manifold lying under DTIs, giving the highest performance among previous DTI prediction methods. However, similarity-based methods are not commonly used at present to predict DTIs, as researchers have found that similarity-based methods work well for DTIs within specific protein classes but not for other classes [7]. In addition, some proteins do not show strong sequence similarity with proteins sharing an identical interacting compound [8].

Thus, feature-based models that predict DTI features of drugs and targets have been studied [9–11]. For feature-based DTI prediction models, a fingerprint is the most commonly used descriptor of the substructure of a drug [12]. With a drug fingerprint, a drug is transformed into a binary vector whose index value represents the existence of the substructure of the drug. For proteins, composition, transition, and distribution (CTD) descriptors are conventionally used as computational representations [13]. Unfortunately, feature-based models that use protein descriptors and drug fingerprints showed worse performance than previous conventional quantitative structure-activity relationship (QSAR) models [9]. To improve the performance of feature-based models, many approaches have been developed, such as the use of interactome networks [14, 15] and minwise hashing [16]. Although various protein and chemical descriptors have been introduced, feature-based models do not show sufficiently good predictive performance [17]. For conventional machine learning models, features must be built to be readable by modeling from original raw forms, such as simplified molecular-input line entry system (SMILES) and amino acid sequences. During transformation, rich information, such as local residue patterns or relationships, is lost. In addition, it is hard to recover lost information using traditional machine learning models.

In recent years, many deep learning approaches have recently been developed and recruited for omics data processing [18] as well as drug discovery [19], and these approaches seem to be able to overcome limitations. For example, DeepDTI built by *Wen et al*. used the deep belief network (DBN) [20], with features such as the composition of amino acids, dipeptides, and tripeptides for proteins and extended-connectivity fingerprint (ECFP) [21] for drugs [7]. The authors also discussed how deep-learning-based latent representations, which are nonlinear combinations of original features, can overcome the limitations of traditional descriptors by showing the performance in each layer. In another study by *Peng et al*. [22], MFDR employed sparse Auto-Encoder (SAE) to abstract original features into a latent representation with a small dimension. With latent representation, they trained a support vector machine (SVM), which performed better than previous methods, including feature- and similarity-based methods. In another study called DL-CPI by *Tian et al*. [23], domain binary vectors were employed to represent the existence of domains used to describe proteins.

One way to reduce the loss of feature information is to process raw sequences and SMILES as their forms. In a paper by *Öztürk et al*., DeepDTA was used to represent raw sequences and SMILES as one-hot vectors or labels [24]. With a convolutional neural network (CNN), the authors extracted local residue patterns to predict the binding affinity between drugs and targets. As a result, their model exhibited better performance on a kinase family bioassay dataset [25, 26] than the previous model, kronRLS [27] and SimBoost [28]. Because their model is optimized by densely constructed kinase affinities, DeepDTA is appropriate to predict kinase affinities not to predict new DTIs with various protein classes. Furthermore, they evaluated their performances on the identical dataset, rather than on independent dataset from new sources or databases.

**Fig 1:**
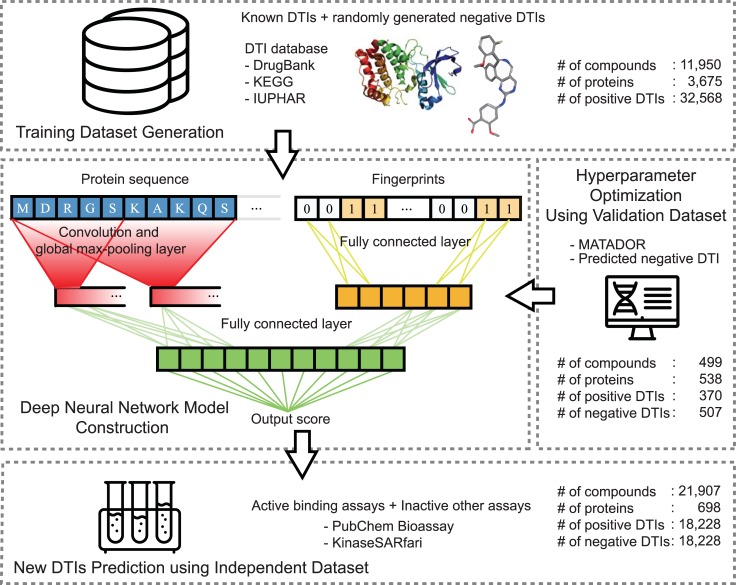
Overview of our model. First, we collected training DTI datasets from various databases (DrugBank, KEGG, IUPHAR). Second, we constructed the neural network model using convolution, which is able to capture local residue patterns that can help the DTIs. Third, we optimized the hyperparameters with an external validation dataset that we constructed. Finally, we predicted DTIs from bioassays (independent test dataset) and evaluated the performance of our model. The numbers (#) of compounds, proteins and DTIs are summarized in each step.

To overcome the aforementioned problems, here, we introduce a deep learning model that predicts massive-scale DTIs using raw protein sequences not only for various target protein classes but also for diverse protein lengths. The overall pipeline of our model is depicted in Fig 1. First, for the training model, we collected large-scale DTIs integrated from various DTI databases, such as DrugBank [29], International Union of Basic and Clinical Pharmacology (IUPHAR) [30], and Kyoto Encyclopedia of Genes and Genomes (KEGG) [31]. Second, in model construction, we adopted convolution filters on the entire sequence of a protein to capture local residue patterns, which are the main protein residues participating in DTIs. By pooling the maximum CNN results of sequences, we can determine how given protein sequences match local residue patterns participating in DTIs. Using these data as input variables for higher layers, our model constructs, abstracts and organizes protein features. After new protein features are generated, our model concatenates protein features with drug features, which come from fingerprints in the fully connected layer and predict the probability of DTIs via higher fully connected layers. Third, we optimized the model with DTIs from MATADOR [32] and negative interactions predicted from *Liu et al*. [33]. Finally, with the optimized model, we predicted DTIs from bioassays such as PubChem BioAssays [34] and KinaseSARfari [35] to estimate the performance of our model. As a result, our model exhibits better performance than previous models.

## Results

### Performances of the validation dataset and selected hyperparameters

**Fig 2:**
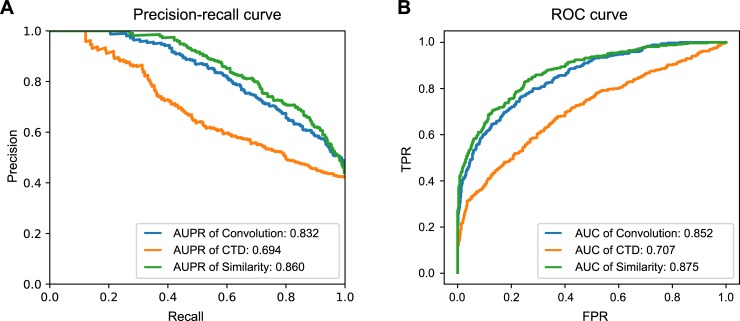
Performance curves for optimized models of protein descriptors. The AUPR and AUC of the convolution, CTD, and similarity descriptors are shown in panels (A) and (B), respectively.

As a normal step of hyperparameter setting, we first tuned the learning rate of the weight update to 0.0001. After the learning rate was fixed, we benchmarked the sizes and number of windows, hidden layers of the drug features, and the concatenating layers with the area under precision-recall (AUPR) on the external unseen validation dataset, which was built with MATADOR and a highly credible negative dataset. Finally, we selected the hyperparameters of the model, as shown in Table A in S1 Text, with the external unseen validation dataset, yielding an AUPR of 0.832 and area under the curve (AUC) of 0.852, as shown in Fig 2. The AUPR value of our model was less than the AUPR of the similarity descriptor; however, that does not mean that our method has lower prediction performance than the similarity method because the size of the validation is too small to evaluate the general performance. In addition, we further examined the effect of fixed maximum protein length on the prediction performance. As shown in Fig A in S1 Text, we confirmed that the prediction performance of our model is not biased to the fixed maximum protein length. Finally, the fully optimized model is visualized as a graph, shown in S1 Fig, respective to our model, the CTD descriptor, and similarity descriptors. In the same manner, we built and optimized models that use other protein descriptors with the same activation function, learning rate, and decay rate.

### Comparison of performance with other protein descriptors

**Fig 3:**
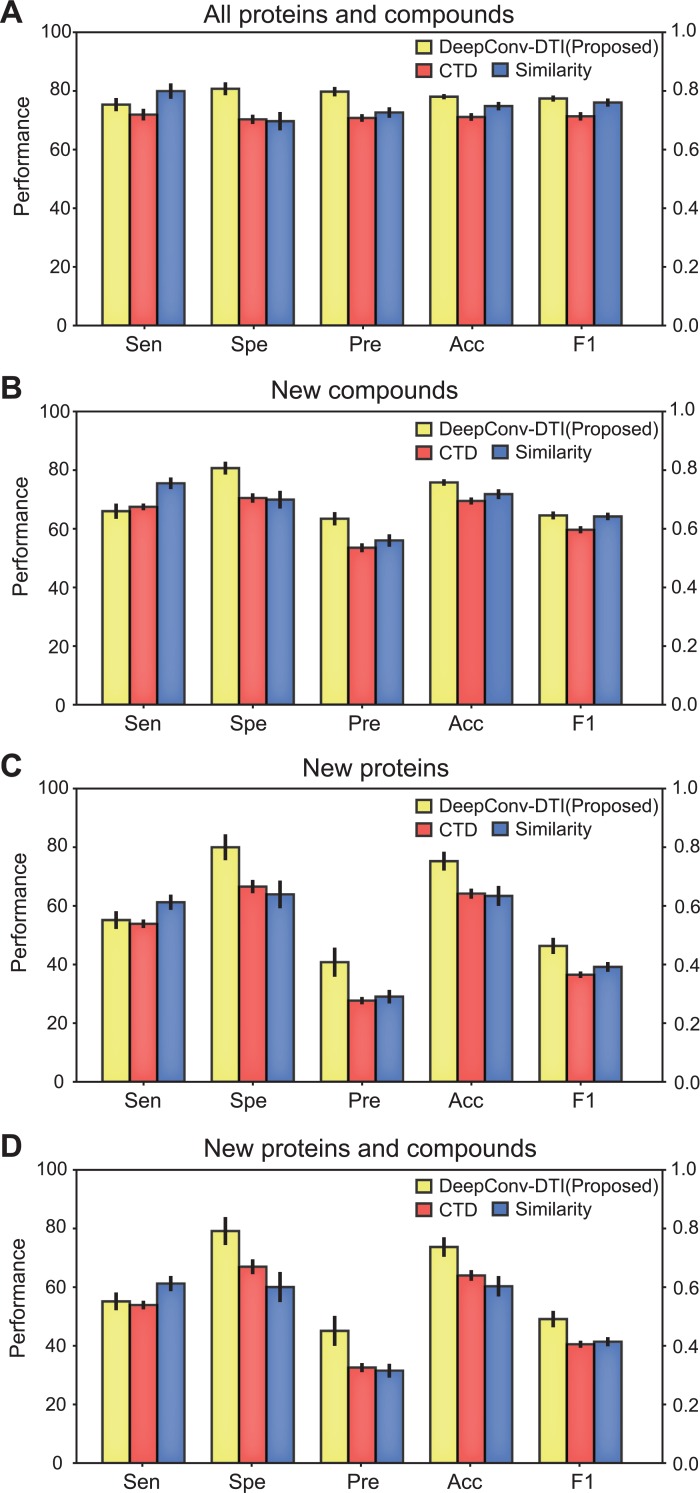
Performance measures for all of the independent datasets of the PubChem dataset. We measured various performances such as sensitivity (Sen.), specificity (Spe.), precision (Pre.), accuracy (Acc.), and F1 score (F1) from the prediction results given by descriptors (A-D). (A) All queried PubChem datasets. (B) PubChem dataset whose compounds are not in the training dataset. (C) PubChem dataset whose targets are not in the training dataset. (D) PubChem dataset whose compounds and targets are not in the training dataset. Our convolution model shows better performances for all datasets in terms of accuracy and F1 score.

After the hyperparameters were tuned, we compared the performance based on the independent test datasets with the different protein descriptors, the CTD descriptor (which is usually used in the conventional chemo-genomic model) [13], the normalized Smith-Waterman (SW) score [36], and our convolution method. The results showed that our model exhibited better performance than the other protein descriptors for all datasets, as shown in Fig 3 and Fig B in S1 Text. With the threshold selected by the equal error rate (EER) [37], our model performed equally well with both the PubChem and KinaseSARfari datasets, indicating that our model has general application power. Our convolution method gave the highest accuracy score and F1 score for the PubChem dataset (Fig 3**A**) [34] and its subsets (Fig 3**B–**3**D**) and a slightly lower F1 score for the KinaseSARfari dataset (Fig B in S1 Text) [35]. The CTD descriptor gave the lowest score for any dataset and any metric, which implies that CTD is less informative and less enriched than the other descriptors. Here, we also observed that the model performance using a similarity descriptor for the KinaseSARfari dataset was similar to that of the proposed model. We can interpret this result as the similarity descriptor acts as an informative feature as a local residue pattern at the domain level, not the whole protein complex.

### Performance comparison with a previous model

In addition to the comparison between convolution in our model and other protein descriptors, in this section, we compared the performance of our model against recently developed deep-learning-based models. We selected three deep learning models for comparisons, SAE (MFDR, *Peng et al*, 2016) [22], DBN (DeepDTI, *Wen et al*, 2017) [7] and CNN (DeepDTA, *Ozturk et al*, 2018). First, MFDR trains SAE in an unsupervised manner, while proteins are represented by multi-scale local descriptor feature [38] and compounds are represented by PubChem fingerprints as input and output for SAE. With trained deep representations of sparse Auto-Encoder, they performed 5-fold cross-validation by using SVM. As a result, their model gives better performances than previous bipartite local models. Because the authors do not provide the model, we implemented the MFDR model with optimized parameters the author provided in their original paper. We tested the validity of implemented MFDR and confirmed that the implemented model produces reasonably same performance compared to the results from its original work (see Fig C S1 Text). Second, DeepDTI built by *Wen et al*. is based on DBN [20], which is a stack of restricted Boltzmann machine (RBM). DeepDTI takes amino acid, dipeptide and tripeptide compositions (protein sequence composition descriptors, PSC) as the protein input and ECFP with radius 1, 2 and 3 as the compound input. We used DeepDTI with the code that the authors provided (https://github.com/Bjoux2/DeepDTIs_DBN) and optimized hyperparameters as the authors mentioned. Third, DeepDTA built by *Ozturk et al*. used stacked CNN on protein sequences and SMILES to predict affinity between target protein and compound. DeepDTA is optimized for Davis [25] and KIBA [26] dataset which contains kinases protein, their inhibitors, and dense affinity values, showing better prediction performances than previous affinity prediction models. We also used DeepDTA with the code from the original work (https://github.com/hkmztrk/DeepDTA) and optimized hyperparameters they provided. For the DTI prediction performance comparison, we activate the last layer with sigmoid function to predict interaction, not affinity, also we changed loss function as binary cross-entropy from mean squared error. It should be noticed that we compared the performance of all three models by training and testing with the same data set we used for a fair comparison.

**Fig 4:**
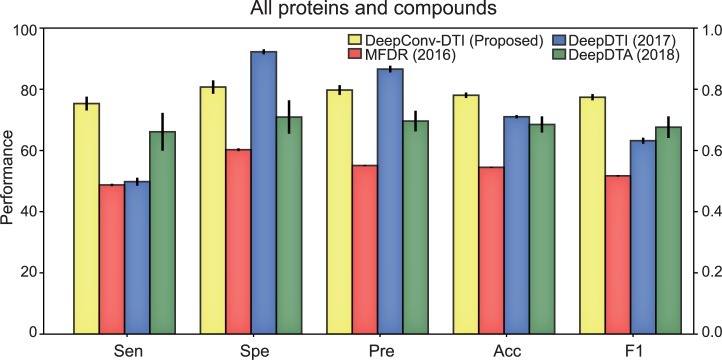
Comparison of performances between our model and previous models. We compared performances of our model on independent test dataset (PubChem) with previous models (MFDR, DeepDTI, and DeepDTA). Our model gives better performances than previous models for accuracy and F1 metrics.

Results of performance comparison between our proposed model and the three related models are shown in Fig 4, showing that performances (accuracy, F1) of our model (DeepConv-DTI) are better than other models. MFDR which gave high AUC in 5-fold cross-validation shows decreased performances in the independent test dataset. We can speculate that SAE which learns deep representation of DTI in an unsupervised way is not appropriate for a case that datasets are composed of various protein classes. In the case of DeepDTI, DeepDTI takes physicochemical properties (PSC) of whole protein sequence including subsequences or domains which do not participate in the interaction with compounds, resulting in worse performance than our model which extracts local residue patterns. For DeepDTA, DeepDTA also shows worse performances than our model with having a relatively large variance. We interpret the worse performance of DeepDTA as follows. DeepDTA is optimized for a densely constructed dataset with specific protein class, while the training dataset in this comparison covers various protein classes (kinase, protease, ion channel, nuclear receptor, GPCR, etc), not only kinase class. Thus, DeepDTA which is specialized for a specific protein class could not achieve better prediction performance in the generalized protein classes.

In addition to the three models we compared, we also compared our model with DL-CPI [23] built by *Tian et al*. which used protein domain information. For proteins whose domain information is not in Pfam [39], datasets for training, validation and test are not fully available. Therefore, we independently compared performances between DL-CPI and our model by additionally built the training, validation, and test datasets. Performance comparison results are described in Fig E in S1 Text. We confirmed that the proposed model shows better performance than DL-CPI. Because protein descriptor of DL-CPI is sparse, containing few values in large dimension, which may decrease performances.

In overall, our model shows better performance than previous deep learning models in an independent test dataset from a different database, which contains distinct DTIs, dealing with DTIs with various protein classes and their interacting compounds.

### Analysis of convolution results

Because we pooled the maximum convolution results by each filter for each window, the pooled results could highlight regions of matches with local residue patterns. Although we cannot measure exactly how those values affect the DTI prediction results, the pooled maximum convolution result will affect the prediction performance by going through higher fully connected layers. Therefore, if our model is capable of capturing local residue patterns, it would give high values to important protein regions, such as actual binding sites.

**Fig 5:**
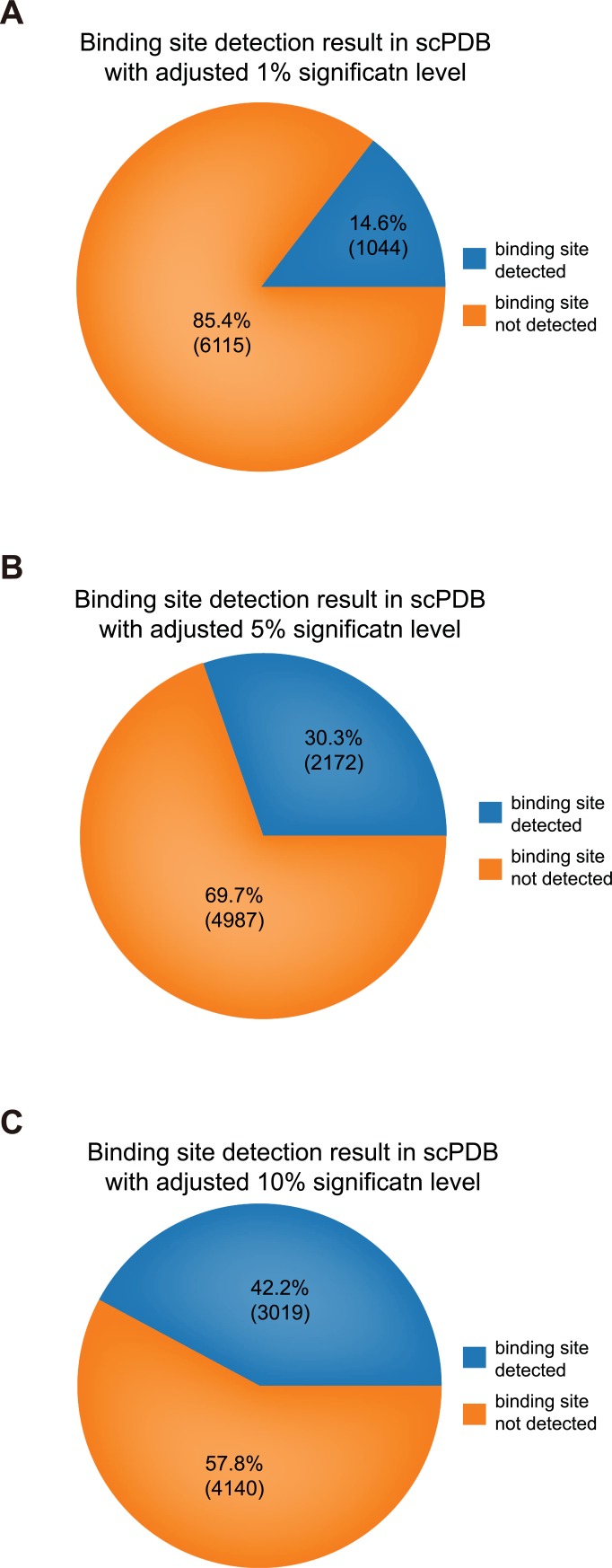
Statistical test for binding region detection. We executed a right-tailed *t*-test for the number of covering binding sites from the convolution results with a null distribution, which was constructed from the randomly generated convolution results in the sc-PDB database consisting of 7,179. Because each sc-PDB test has many windows, we selected the most significant p-values adjusted by the Benjamini-Hochberg procedure and examined whether they were significant at levels of 1%, 5% and 10%. The results showed that 14.6%, 30.3% and 42.2% of sc-PDB entries were significantly enriched, respectively (A-C).

**Fig 6:**
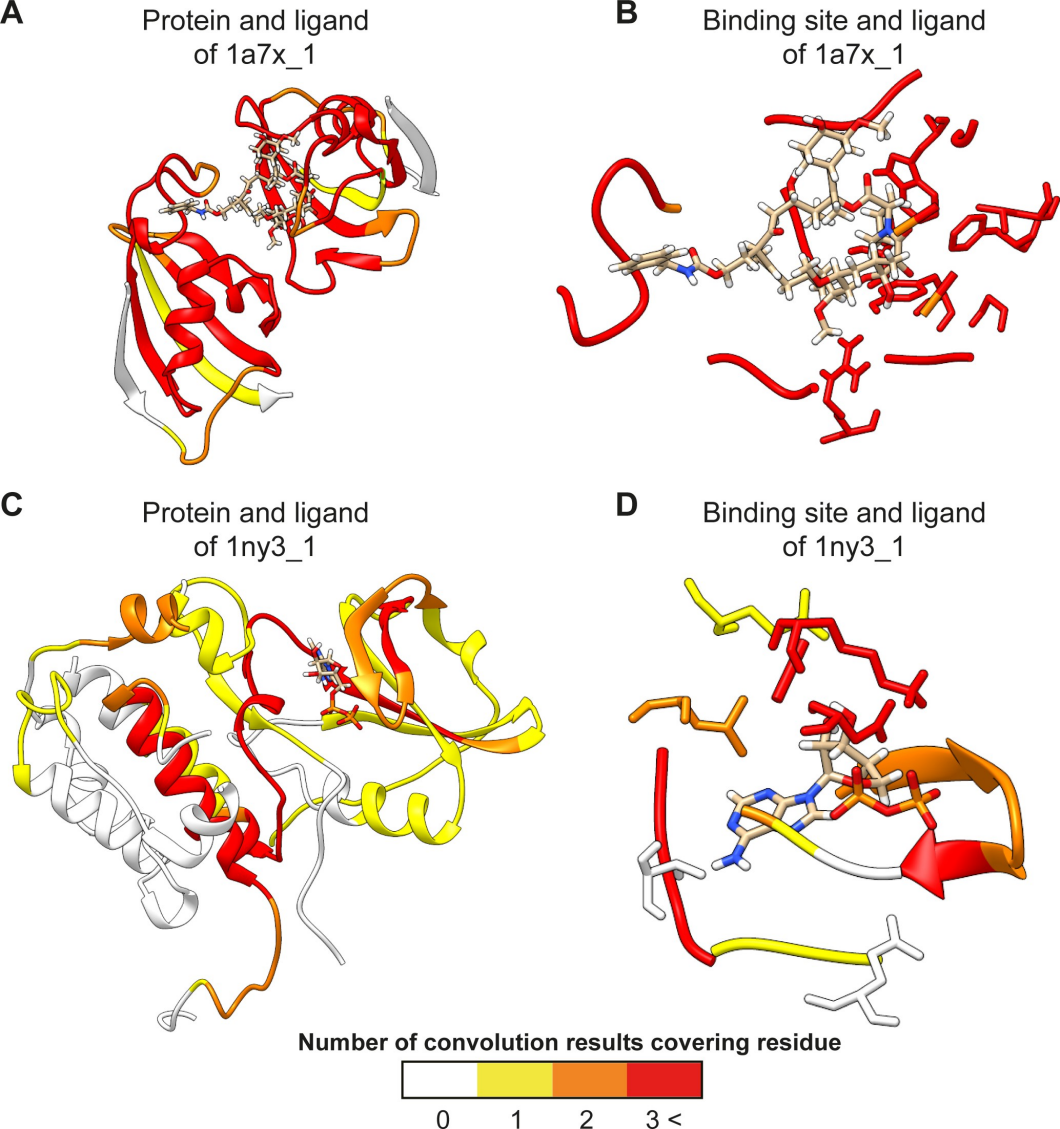
Visualization of convolution results. We visualized two highly scored sc-PDB entries from two perspectives—the whole receptor-ligand complex and binding site-ligand perspectives—by using UCSF Chimera. To visualize convolution results with a simplified view, first, we selected the top 5 ranked globally max-pooled results among all filters for each window because whole protein sequences are usually covered by convolution results if we select all results. Second, we rendered residues covered by the convolution results by the number of covering convolution results. (A) Complex of the ion channel protein Peptidyl-prolyl cis-trans isomerase FKBP1A (FKB1A_HUMAN in UniProt), which has a short sequence length (108), and FKA in the PDB ligand (1a7x_1 in sc-PDB). As we can see, the number of convolution results near the ligand is more than the number for the other region. (B) For the binding site and ligand of 1a7x_1, most of the binding sites are highly covered by the convolution results. (C) Complex of the kinase protein MAP kinase-activated protein kinase 2 (MAPK2_HUMAN in UniProt) with a sequence length of 400 and ADP in the PDB ligand (1ny3_1 in sc-PDB). Although half of the protein sequence is not represented as a 3D structure, our convolution results cover regions close to ligand binding sites in a biased manner. However, some residues far from binding sites are also highlighted by convolution results, potentially indicating some important structural motifs for binding. (D) For the binding site and ligand of 1ny3_1, most binding sites are covered by the convolution results, although some residues are not covered.

Examining and validating the convolution results from the intermediate layer showed that our model could capture local residue patterns that participate in DTIs. The sc-PDB database provides atom-level descriptions of proteins, ligands, and binding sites from complex structures [40]. By parsing binding site annotations, we can query binding sites between protein domains and pharmacological ligands for 7,179 entries of Vertebrata. From the queried binding sites and pooled maximum convolution results, we statistically test our assumption that the pooled maximum convolution results cover the important regions, including binding sites. Each window has 128 pooled convolution results, which shows bias in covering some regions. Thus, we randomly generated 128 convolution results 10,000 times for each sc-PDB entry and counted how many of those random results covered each amino acid in the binding sites, which resulted in the construction of normal distributions. For each normal distribution constructed by the randomly generated convolution results, considered a null hypothesis, we executed a right-tailed *t*-test with the number from the convolution results of our model for each window. Because we did not know which window detects the binding site, we took the most significant p-value (minimum p-value adjusted by the Benjamini-Hochberg procedure [41]). The sc-PDB entry information and p-values of a window for each sc-PDB entry are summarized in the S1 File. We summarize the results of binding site detection from the most significant p-value among windows by significance level cutoff in Fig 5. In addition, we examined sc-PDB entries with the most significant p-values for diverse window sizes. We visualized two high-score sc-PDB entries from two perspectives—the whole receptor-ligand complex and binding site-ligand perspectives—by using UCSF Chimera [42] as shown in Fig 6. To visualize convolution results with a simplified view, first, we selected the top 5 ranked globally max-pooled results among all filters for each window because whole protein sequences are usually covered by convolution results if we select all results. Second, we rendered residues covered by convolution results by the number of covering convolution results. We visualized two sc-PDB entries, 1a7x_1 and 1ny3_1. 1a7x_1, representing the complex of the ion channel, protein Peptidyl-prolyl cis-trans isomerase FKBP1A (FKB1A_HUMAN in UniProt), which has a short sequence length (108), and BENZYL-CARBAMIC ACID [8-DEETHYL-ASCOMYCIN-8-YL]ETHYL ESTER (FKA in PDB ligand) [43]. 1ny3_1 is the complex of the kinase protein, MAP kinase-activated protein kinase 2 (MAPK2_HUMAN in UniProt) with sequence length 400, and ADENOSINE-5’-DIPHOSPHATE (ADP in PDB ligand) [44]. Through the above evaluation, we can confirm that our proposed model is capable of capturing local residue patterns of proteins that are considered important features for DTI prediction, such as actual binding sites.

### t-SNE visualization of proteins

From the results shown in Fig 6, we can confirm that our model can capture the local residue patterns of proteins that participate in DTIs. Thus, to examine further characteristics of the captured protein local residue patterns, we visualized the protein features from the fully connected layer after the global max-pooling of convolution results. We visualized 1,527 proteins used in the training dataset categorized in various protein classes. Specifically, we visualized 257 GPCRs, 44 nuclear receptors, 304 ion channel receptors, 604 kinases, and 318 proteases. For visualization, we conducted t-distributed stochastic neighbor embedding (t-SNE) for dimension reduction and visualization [45]. t-SNE can map high-dimensional features to low-dimensional ones, such as 2-dimensional features, minimizing information loss during dimension reduction. Surprisingly, although our model is not intended to identify protein classes, it can roughly discriminate protein classes from the intermediate protein layer, as shown in Fig G in S1 Text.

## Discussion

In this work, we built a novel DTI prediction model to extract local residue patterns of whole target protein sequences with CNN. We trained the model with DTIs from various drug databases and optimized the model with an external validation dataset. As a result, the detected local features of protein sequences perform better than other protein descriptors, such as CTD and SW scores. Our model also performs better than a previous model built on DBN. In addition, by analyzing pooled convolution results and statistically and manually comparing them with annotations from sc-PDB entries, we showed that, for some proteins, our model is capable of detecting important regions, including binding sites. Therefore, our approach of capturing local residue patterns with CNN successfully enriches protein features for DTI prediction.

The number of 3D structures in Protein Data Bank [46] is relatively smaller than the number of sequences, limiting 3D structure-based DTI prediction methods. For example, the number of PDB entries for *Homo sapiens* is 42,745, while the number of protein sequences for *Homo sapiens* is 177,661 in UniProtKB. However, our method does not depend on the 3D structure of proteins because it considers only protein sequence, rather than classical protein feature descriptors such as the CTD descriptor and normalized SW score. As a result, our method can be more generally applied to predict DTIs than methods needing 3D structures.

Although our model shows improved prediction performance, there is still room for improvement. First, we simply used Morgan/Circular fingerprints, which are binary and have large dimensions. Therefore, we will use more informative chemical descriptors, based on neural networks for DTI prediction, to achieve advanced performance. Second, as shown in a previous study [47], considering 3D structure information is an effective substitution for chemical elaboration. Therefore, in the future, we will elaborate upon our model by considering 3D structure features.

## Materials and methods

### Building dataset

To build the training dataset, we obtained known DTIs from three databases: DrugBank, KEGG, and IUPHAR. To remove duplicate DTIs among the three databases, we unified the identifiers of the compounds and the proteins. For the drugs, we standardized the identifiers of the compounds in the DrugBank and KEGG databases with the InChI descriptor. For the proteins, we unified the identifiers of the proteins as UniProtKB/Swiss-Prot accessions [48]. Among the collected DTIs, we selectively removed proteins of Prokaryota and single-cell Eukaryota, retaining only proteins of Vertebrata. Finally, 11,950 compounds, 3,675 proteins, and 32,568 DTIs were obtained in total. Because all collected DTIs are regarded as positive samples for training and negative DTIs are not defined in the databases above, a random negative DTI dataset is inevitably generated. To reduce bias from the random generation of negative DTIs, we built ten sets of negative DTIs exclusively from the positive dataset. The detailed statistics of the collected training dataset are shown in Table D in S1 Text.

To optimize our model with the most adequate hyperparameters, we constructed an external validation dataset that had not seen DTIs in the training phase. We collected positive DTIs from the MATADOR database [32], including ‘DIRECT’ protein annotations, and all DTIs observed in the training dataset were excluded. To build a credible negative dataset, we obtained negative DTIs via the method of *Liu et al*. [33]. This method selects candidate negative DTIs with low similarity to known positive DTIs. From the obtained negative dataset, we balanced the negative dataset with the positive dataset, using a negative score (>0.95). As a result, 370 positive DTIs and 507 negative DTIs were queried for the external validation set. The statistics of the external validation dataset are summarized in Table E in S1 Text.

To evaluate our model, we built two independent test datasets from the PubChem BioAssay database [34] and ChEMBL KinaseSARfari [35]; these datasets consisted of results from experimental assays. To obtain positive DTIs from PubChem, we collected ‘Active’ DTIs from the assays with the dissociation constant (K_d_ < 10μ*m*) [49]. Because we sought to predict whether a drug binds to a protein, among the many types of assays (Potency, IC_50_, AC_50_, EC_50_, K_d_, K_i_), evaluation of the dissociation constant (K_d_) was the most appropriate assay for obtaining positive samples. For the negative samples, we took the samples annotated as ‘Inactive’ from the other assay types. Because there were too many negative samples in the PubChem BioAssay database, we first collected only negative samples whose drug or target was included in the positive samples from the PubChem BioAssay database. Second, we selected as many random negative samples as positive DTIs from PubChem BioAssay. As a result, total 36,456 positive and negative samples were built with 21,907 drugs and 698 proteins. For the performance evaluation, we created three subsets of the PubChem bioassay independent dataset for humans, which consisted of only new compounds, new proteins, and new DTIs. Detailed summaries of the PubChem dataset and its subset are shown in Table F in S1 Text. We also collected samples from KinaseSARfari. KinaseSARfari consists of assays involving a compound that binds to a kinase domain. To obtain positive samples from KinaseSARfari, we considered each assay result with a dissociation constant of (K_d_ < 10μ*m*) as positive [49]; this value is sufficiently small to be considered positive. In contrast to the PubChem BioAssay, the number of negative samples was similar to the number of positive samples in KinaseSARfari; therefore, we did not sample the negative samples. We collected 3,835 positive samples and 5,520 negative samples with 3,379 compounds and 389 proteins. Detailed statistics of the KinaseSARfari dataset are shown in Table F in S1 Text. In addition, we summarize the portion of the protein class in each dataset in Fig H in S1 Text. Here, we confirmed that the training and the validation datasets were not biased toward a specific protein class.

### Drug feature representation

In our model, we used the raw protein sequence as the input for the protein but did not use the raw SMILES string as the input for the drug. For the drug, we used the Morgan/Circular drug fingerprint, which analyzes molecules as a graph and retrieves substructures of molecular structures from subgraphs of the whole molecular graph [21]. Specifically, we used RDKit [50] to yield a Morgan/Circular fingerprint with a radius of 2 from a raw SMILES string. Finally, each drug can be represented as a binary vector with a length of 2,048, whose indices indicate the existence of specific substructures.

### Deep neural network model

#### Overall schema of the deep learning network

We extracted the local residue patterns from protein sequences via CNN and yielded a latent representation of drug fingerprints via fully connected layers. After processing both the drug and protein layers, we concatenated these layers and constructed the fully connected layer, resulting in the output. Every layer except the output layer was activated with the exponential linear unit (ELU) function [51].

$$\sigma (\alpha ,x)=\{\begin{array}{cc} \alpha \left(e^{x}-1\right) & \mathrm{for}\;x<0\\ x & \mathrm{for}\;x\geq 0 \end{array}$$

The output layer was activated with the sigmoid function for classification. The whole neural network model was implemented with Keras (2.16) [52].

#### Convolution layer with protein embedding vector

**Fig 7:**
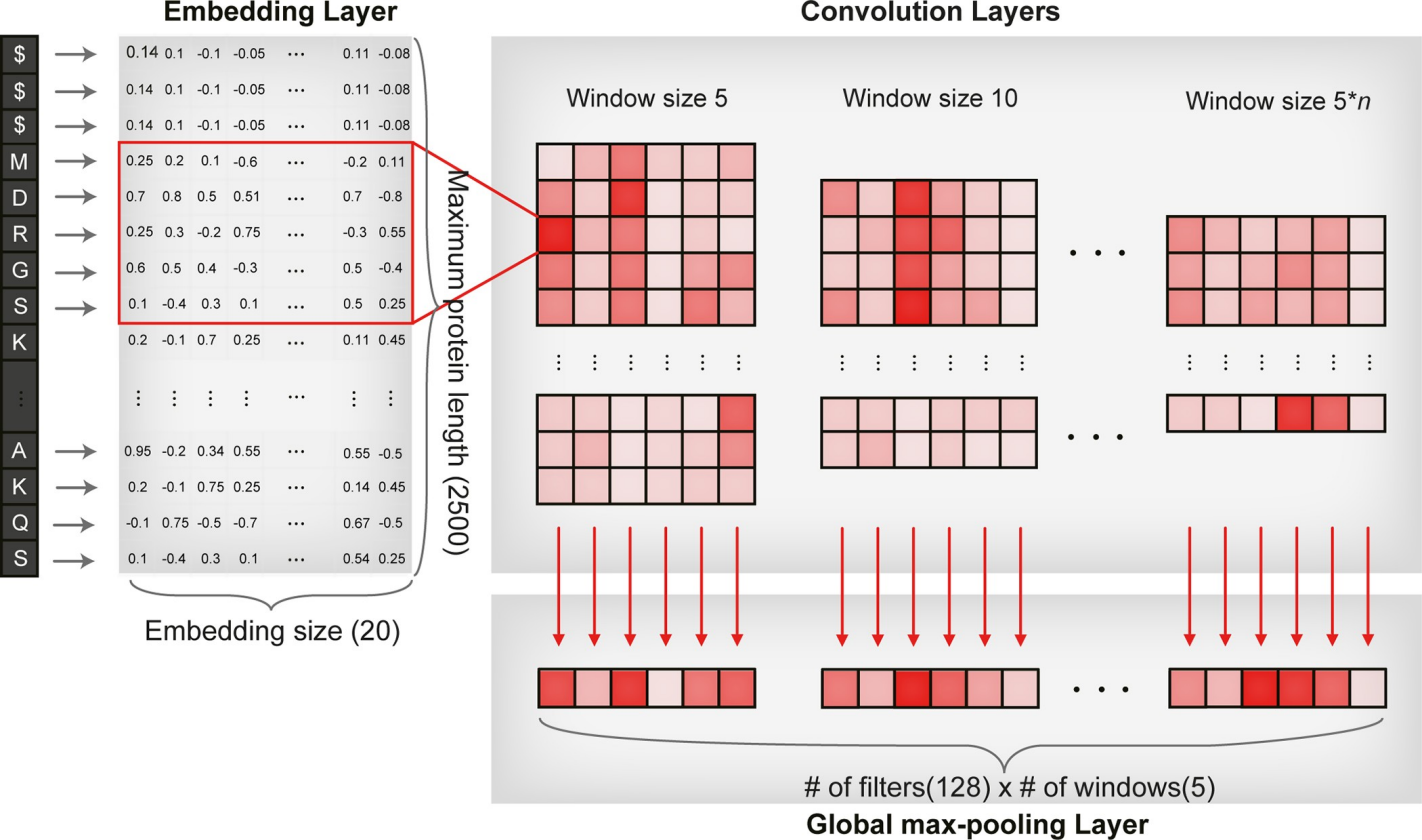
Overall schema for extracting the local patterns from the whole protein sequence. First, we transformed the protein sequence to an embedding vector with a fixed size, and the margins were padded, which are marked as $ and the corresponding embedding vectors. Second, we executed convolution along the sequence. Third, for each filter of window size, we pooled the max value. By concatenating all of the max-pooling values, we built a protein feature vector whose dimension is multiplying the number (#) of filters by the number (#) of windows.

One of the difficulties in describing the protein features for the machine learning model and the deep learning model was that the protein lengths were all different. Another difficulty was that only certain parts of a protein, such as specific domains or motifs, are involved in DTIs, rather than the whole protein structure. As a result, the physicochemical properties of the whole protein sequence do not seem to be appropriate features for predicting DTIs due to noise information from the portions of the sequence that are not involved in the DTIs. Thus, the extraction of local residue patterns involved in the DTIs is necessary for precise prediction, and CNN is known to capture important local patterns from the whole space. The overall schema of convolutional layers is depicted in Fig 7. The model starts with an embedding to transform each amino acid to the corresponding embedding vector. The embedding layer is a lookup table of embedding vectors. Embedding vector values are randomly initialized by the Xavier initializer (denoted ‘glorot normal’ in keras), which imposes normal distribution of weights and variance of output following variance of input [53]. Embedding vectors are trainable, meaning that embedding vector values are also changed to optimize loss during training. From the lookup table, the embedding matrix for the protein sequence is constructed by querying embedding vectors corresponding to amino acids from the embedding layer, as described in Fig I in S1 Text. The length of the embedding matrix for all proteins was set to the same as the maximum protein length, i.e., 2,500, and the margins were padded with null labels ($) and the corresponding embedding vectors, which would give a meaningless convolution result that is filtered out during global max-pooling as depicted in Fig J in S1 Text. As a result, an embedding layer was constructed for protein features. We executed convolution on the embedding layer of the protein along the sequence in 1D fashion with striding 1, with convolution from j^th^ the to the (j+WS)^th^ amino acids in sequence, which can be defined as

$${\left(x*w\right)}_{j}=\sum_{a=1}^{ES}\sum_{b=0}^{WS-1}w_{a,b}x_{a,j+b}$$

Convolution for the whole sequence results in a (MPL-WS+1) size convolution layer for each filter, where WS is the window size. Finally, to extract the most important local feature, we conducted global max-pooling for each filter, which is defined as

$$MaxPooling_{global}(E_{p_{k}})=max((x*w{)}_{j})$$

where j covers all of the convolution results of the embedding matrix from protein sequence p_k_, resulting in a filter-sized vector with a max-valued convolution result for each window, which does not induce bias from the locations of local residue patterns and the maximum protein length. After pooling all convolution results, we concatenated them to represent the important local patterns for interactions as a vector-formatted feature. Finally, for the organization and abstraction of protein features, concatenated max-pooling results are fed into fully connected layers, which constructs a latent representation of protein.

#### Fully connected layers for drug fingerprints and concatenating layer

As mentioned in the Introduction, latent representations of the drug fingerprint descriptors made by fully connected layer are useful for predicting DTIs. After features of the protein and drug were refined by the neural network, we concatenated them and constructed fully connected layers to predict whether the drug and target interact.

#### Calculation of loss and weight optimization

Using the constructed deep neural model, the input flows to the output layer in a feed-forward fashion. The deep neural model calculates loss with binary cross-entropy:

$$J(W,b)=-\frac{1}{n}\sum_{i}^{n}\left[y_{i}\;\log\hat{y_{i}}+(1-y_{i})\;\log\left(1-\hat{y_{i}}\right)\right]$$

To prevent overfitting, we penalized the loss function with L2-norm:

$$J_{L2}(W,b)=J(W,b)+\lambda \sum_{l=1}^{L-1}{\left\|W^{l}\right\|}_{2}$$

Finally, we updated the weights using the Adam optimizer [54] with a penalized loss to give a generalized prediction for the model.

#### Regularizations of the neural network

In the artificial neural network technique, there are several ways to prevent overfitting. Currently, dropout and batch normalization are most frequently used to regularize neural networks. Dropout masks hidden nodes in the training phase, which makes a subset of hidden nodes unavailable to predict results for training labels [55]. By masking some hidden nodes in training, dropout generalizes the model, making the model independent of a specific dataset. We used 1-dimensional spatial dropout on the embedding layer [56]. In addition, we used a batch normalization technique to prevent overfitting except in the embedding layer. Batch normalization normalizes the outputs of the neural network with a mean of 0 and a standard deviation of 1 on a minibatch. However, batch normalization could induce a loss in the influence of parameters and linearity of network outputs, rather than nonlinearity. Thus, batch normalization induces a scale factor and a shift factor for normalized outputs, whose values are also introduced in the learning phase, to resolve the problem [57].

#### Selection of hyperparameters

In our deep learning models, hyperparameters, such as the learning rate and window sizes that affect performance, are tuned during cross-validation. However, the hyperparameters should not be determined based on the performance of the subset of the training dataset because the negative datasets are randomly sampled. With the external validation dataset, we first determined the learning rate because a model with a high learning rate is unable to learn a pattern. After the learning rate was selected, we selected activation function and regularization parameters such as the dropout ratio. Finally, we employed a grid-search method for optimization of the other hyperparameters that determine neural network shape. The search range of optimization is summarized in Table A in S1 Text. We identified hyperparameters that exhibited the best AUPR, which is an appropriate performance evaluation metric for the accuracy of classifying the positive sample. The other descriptors to compare with our methods are numerical vectors, which do not have locality. Therefore, we put fully connected layers on the protein descriptors. We also employed a grid-search strategy while sustaining hyperparameters not related to model shape. When the AUPR is measured, the optimal threshold can be given by the EER [37].

$$EER=\underset{\theta }{\mathrm{argmin}}\left(|1-recall|-\gamma |1-precision|\right)$$

where θ is the classification threshold and γ is the constant determining the cost ratio for misclassification from precision and recall, which is set at 2 in our model.

### Sparse Auto-Encoder (SAE) construction

SAE is Auto-Encoder whose distribution of latent representations is regularized with sparsity term [58]. In loss calculation, Kullback-Leibler divergence (KLD) loss between Bernoulli distributions each dimension in latent representation $\hat{\rho }$ and desired sparsity parameter ρ is added to reconstruction loss of Auto-Encoder and ridge loss for weights.

$$J_{sparse}(W,b)=J(W,b)+\beta \sum_{j}^{s_{2}}KL(\rho ||{\hat{\rho }}_{j})$$

where

$$\hat{\rho_{j}}=\frac{1}{m}\sum_{i=1}^{m}\left[a_{j}^{\left(2\right)}(x^{\left(i\right)})\right]$$

During the training of the neural network, KLD acts as a constraint for latent representation following desired sparsity parameter. As a result, for each dimension of latent representation, only a few samples are activated, giving a more reliable representation of original input. In the previous study, MFDR used SAE to build an informative latent representation of DTI, which are composed of multi-scale local descriptors [38] and PubChem fingerprints.

### Deep belief network (DBN) construction

DBN is a generative graphical model proposed by Geoffrey Hinton [20]. DBN is actually a stack of an RBM. RBM consists of visible and hidden units, constructing a bipartite graph. In RBM, probabilistic distribution of visible units is learned in an unsupervised way, with a probabilistic distribution of visible and hidden units

$$P(v,h|W)=\frac{1}{Z}e^{a^{T}v+b^{T}h+v^{T}Wh}$$

and marginal distribution of visible units

$$P(v|W)=\frac{1}{Z}\sum_{h}e^{a^{T}v+b^{T}h+v^{T}Wh}$$

to maximize the probability of visible units for V in a training set with weight matrix W

$$\underset{W}{\mathrm{argmax}}\prod_{v\in V}P(v|W)$$

In DBN, during stacking of RBMs, hidden units of the previous RBM are fed as visible layers of the next RBM. In addition, RBM adopts contrastive divergence for fast training, which uses gradient descent and Gibbs sampling. In a previous study, DeepDTI, the input concatenation of drug and target protein features, PSC descriptors and ECFP with a radius of 1, 2 and 3, was considered a first visible layer. The authors attached logistic regression to the last hidden units to predict DTIs.

### Evaluation of performances

To measure the prediction performance of our deep neural model based on the independent test dataset after the classification threshold was fixed, we obtained the following performance metrics: sensitivity (Sen.), specificity (Spe.), precision (Pre.), accuracy (Acc.), and the F1 measure (F1). See the formulas below:

Sen.=TP/P

Spe.=TN/N

Pre.=TP/(TP+FP)

Acc.=(TP+TN)/(P+N)

F1=(Sen*Pre)/(Sen+Pre)

where TP is true positive, TN is true negative, FP is false positive, FN is false negative, T is positive, and N is negative.

## Supporting information

S1 Text Supporting information. (PDF)

S1 Fig Graph visualization of optimized models. (PDF)

S1 File Metadata and results of statistical test for sc-PDB entries. (CSV)
