# Supplementary material for: DeepConv-DTI: Prediction of drug-target interactions via deep learning with convolution on protein sequences
Source: PLoS Comput Biol. 2019 Jun 14;15(6):e1007129. doi: 10.1371/journal.pcbi.1007129 (PMC6594651; doi:10.1371/journal.pcbi.1007129)
Supplement: S1 Text — (PDF) [file pcbi.1007129.s001.pdf]

DeepConv-DTI: Prediction of drug-target interactions via deep learning with convolution on protein sequences

Ingoo Lee¶, Jongsoo Keum¶, and Hojung Nam*

School of Electrical Engineering and Computer Science, Gwangju Institute of Science and Technology, 123 Cheomdangwagi-ro, Buk-ku, Gwangju, 61005, Republic of Korea

¶Equally contributing authors

*Corresponding author

E-mail: hjnam@gist.ac.kr

S1 Text

Model validation and performance evaluations 4

Table A. Search range and selected values of hyperparameters for the deep learning model 6

Fig A. Performance comparison between different maximum protein lengths. 7

Fig B. Performance tests on the KinaseSARfari dataset 8

Fig C. Performance comparison on *Yamanishi et al.* [5] datasets between original MFDR and implemented MFDR 9

Performance Comparison between the proposed model and DL-CPI 10

Table B. Statistics of reconstructed datasets 12

Table C. Selected hyperparameters of implemented DL-CPI 12

Statistics and protein class ratios for datasets 15

Fig G. t-SNE results with protein features from the fully connected layer beyond the global max-pooling layer of the convolution result. 16

Table D. Statistics of the training dataset 17

Table E. Statistics of the external validation dataset 17

Table F. Statistics of the independent dataset 18

Fig H. Percentages of protein classes in the training, validation, and test datasets. 19

Architecture of the convolutional neural network for protein feature representation 21

Fig I. Lookup table mechanism in the embedding layer. 22

Fig J. Global max-pooling on protein sequence and its embedding layer. 23

References...24

Model validation and performance evaluations

In our deep learning models, hyperparameters, such as the learning rate and window sizes that affect performance, are tuned during cross-validation. However, the hyperparameters should not be determined based on the performance of the subset of the training dataset because the negative datasets are randomly sampled. With the external validation dataset, we first determined the learning rate because a model with a high learning rate is unable to learn a pattern. After the learning rate was selected, we selected activation function and regularization parameters such as the dropout ratio. Finally, we employed a grid-search method for optimization of the other hyperparameters that determine neural network shape. The search range of optimization and selected hyperparameters are summarized in Table A. In addition to validation step, we compared effect on performance for different protein lengths. As shown in Fig A, we confirmed that the prediction performance of our model is not biased to the fixed maximum protein length.

We compared performance based on independent test datasets with different protein descriptors, the CTD descriptor (which is usually used in the conventional chemo-genomic model) [1], normalized SW score [2], and our convolution method by calculating various performance metrics such as sensitivity (Sen.), specificity (Spe.), precision (Pre.), accuracy (Acc.) and F1 score (F1).

The results showed that our model exhibited better performance than the other protein descriptors for all datasets, as shown in Fig 3 and Fig B in S1 Text. Our model performed equally well with both the PubChem and KinaseSARfari datasets, indicating that our model has general application power. Our convolution method gave the highest accuracy score and

F1 score for the PubChem dataset (Fig 3A) [3] and its subsets (Fig 3B-D) and a slightly lower F1 score for the KinaseSARfari dataset as shown in Fig B [4]. The CTD descriptor gave the lowest score for any dataset and any metric, which implies that CTD is less informative and less enriched than the other descriptors. Here, we also observed that the model performance using a similarity descriptor for the KinaseSARfari dataset was similar to that of the proposed model as shown in Fig. We interpret this result as showing that the similarity descriptor acts as an informative feature as a local residue pattern at the domain level, not the whole protein complex.

Table A. Search range and selected values of hyperparameters for the deep learning model

Hyperparameter	Search range	Selected value
**Learning rate**	[0.01, 0.001, 0.0001]	0.0001
**Decay rate**	[0.01, 0.001, 0.0001]	0.0001
**Dropout ratio**	[0, 0.2, 0.5]	0
**Spatial 1D dropout ratio on embedding layer**	[0, 0.2, 0.5]	0.2
**Threshold**		0.2
**Epoch**	0~40	15
**Activation function**	[Sigmoid, ReLU, ELU]	ELU
**# of filters**	[32, 64, 128]	128
**Protein windows**	[5, 10, 15, 20, 25, 30, 35]	10, 15, 20, 25, 30
**Fully connected layers for the protein**	[128, 64]	128
**Fully connected layers for the drug**	First layer: [1024, 512, 256]<br>Second layer: [256, 128, 64]	512,128
**Fully connected layers after concatenating layers**	Frist layer: [256, 128, 64]<br>Second layer: [64, 32]	256

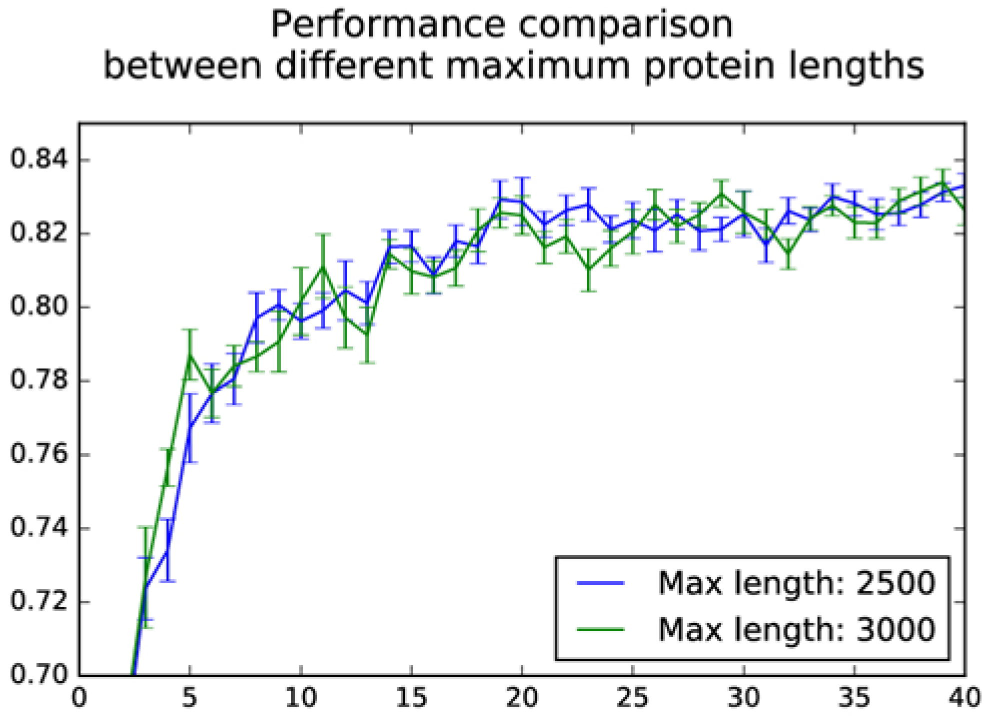

Fig A. Performance comparison between different maximum protein lengths.

We benchmarked performance by maximum protein length along epochs. As expected, maximum protein length does not affect the overall AUPR in the validation dataset.

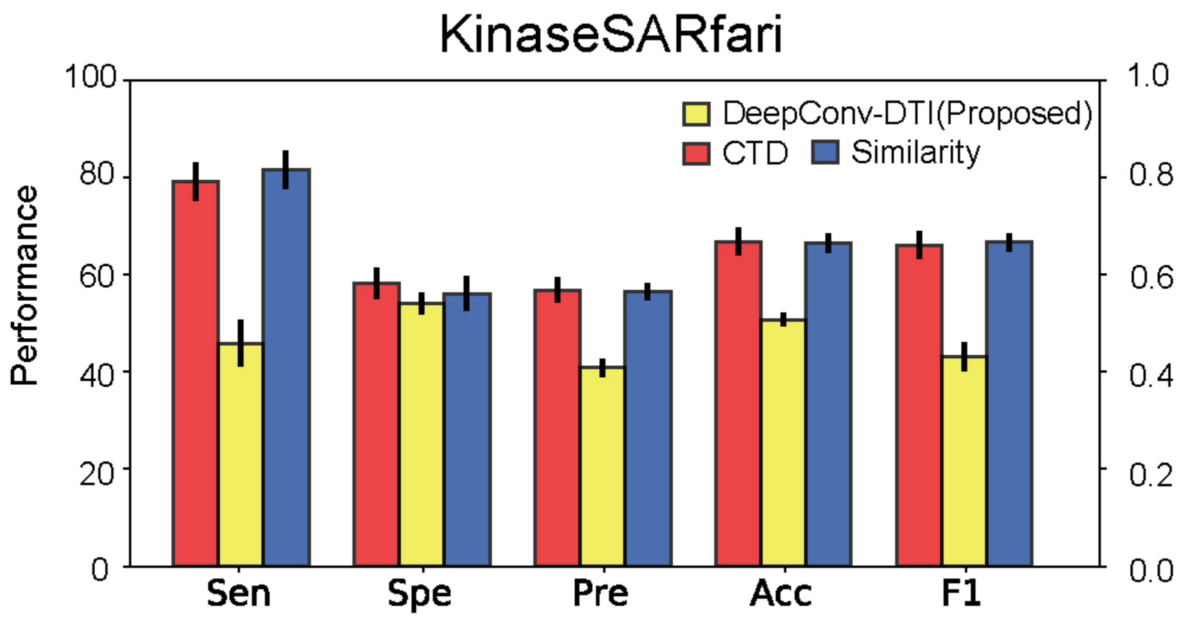

Fig B. Performance tests on the KinaseSARfari dataset.

The KinaseSARfari dataset consists of bioassays with kinase domains and compounds. For the KinaseSARfari dataset, our model gives similar performance to the similarity descriptor.

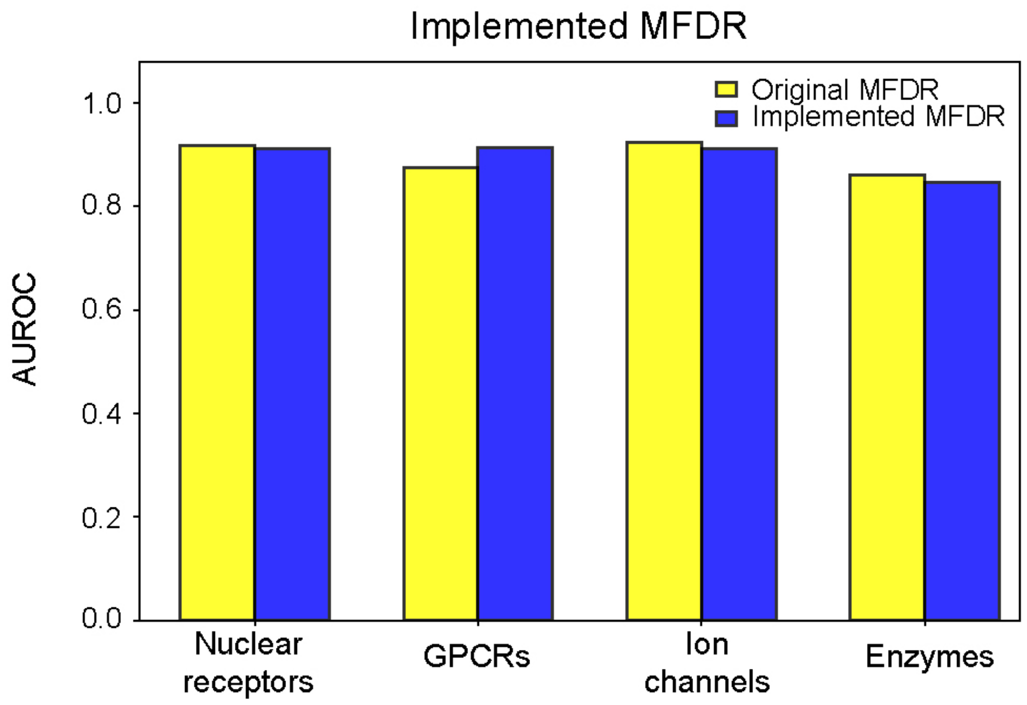

Fig C. Performance comparison on *Yamanishi et al.* [5] datasets between original MFDR and implemented MFDR

We implemented MFDR [6] with the 4-bit binary form of protein descriptor [7] to build a deep representation of DTIs in an unsupervised way. We executed 5-fold cross-validation on *Yamanishi et al.* dataset as its authors did. As a result, the area under the curve on receiver operating characteristics (AUROC) for dataset gives similar performances with authors showed. Specifically, AUROCs of implemented MFDR are 0.846, 0.914, 0.911 and 0.913 and original MFDR are 0.862, 0.875, 0.924 and 0.919, respect to nuclear receptors, GPCRs, ion channels and enzymes.

Performance Comparison between the proposed model and DL-CPI

DL-CPI built by *Tian et al.* [8] yields protein domain information as a descriptor. Protein are represented as a binary vector whose indices indicate the existence of specific domain, and compounds are represented by PubChem fingerprints. DL-CPI stacks fully-connected layers with relu activation for the latent representation of DTI. The more input DTI feature passes layer, the better latent DTI representations are separated in latent space, by positive/negative labels. Because the number of total unique protein domains varies by training dataset, the input dimension of DTI differs by training dataset, which means that optimized hyperparameters of DL-CPI does not work for our training dataset. To compare our proposed model and DL-CPI, firstly we parsed domain information of our training proteins from Pfam [9]. As a result, 1,841 proteins from 3,675 training proteins have 958 domains totally, as shown in Fig D, which implies that the input dimension of implemented DL-CPI model will be 1,839 (958+881). We reconstructed our datasets with proteins which has domain information in training dataset. Statistics of datasets are summary below in Table B. Secondly, we optimized hyperparameters with given ranges that authors suggested in the paper. Selected hyperparameters are summarized in Table C. Specifically, stochastic gradient decent optimizer (SGD) does not show high performances, while Adam optimizer [10] shows much higher than SGD optimizer, which means that there are many small local optima which are hard to overcome for SGD. For our model, we used same hyperparameters, shown in Table A. Optimized performances on validation dataset of models in Fig E. Finally, we compare performances between our proposed model and DL-CPI shown in Fig E. Our model shows better performance than DL-CPI overall. Protein input of DL-CPI is very sparse, containing few values in large dimensions. However it shows good enough performance, especially F1 for the test dataset. We examined protein

classes in datasets, and statistics of protein classes in datasets is summarized in Fig F. Ratio of kinase proteins increases, compared with original ratio shown in Fig H, which means that their domain information is well figured out, making performances of simple deep learning model relatively better.

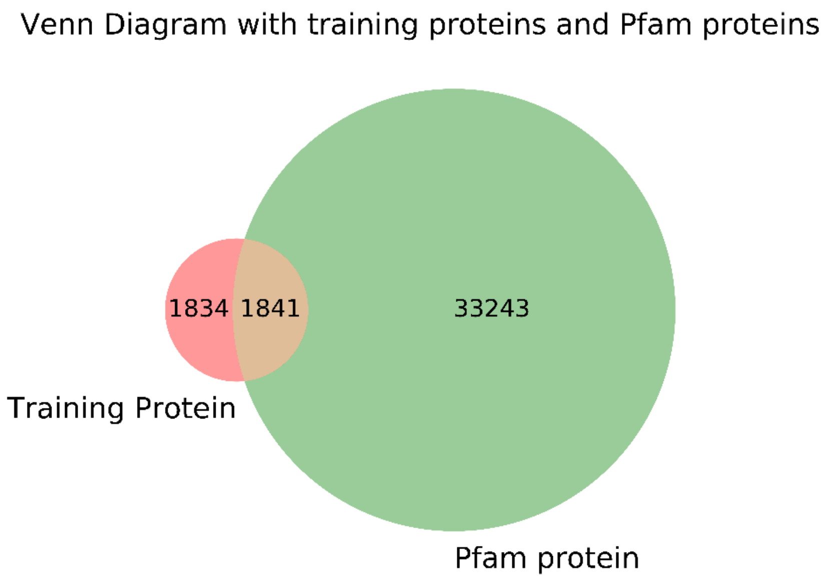

Fig D. Venn diagram with training proteins and Pfam proteins.

We parsed domain information of our training proteins from Pfam. As a result, 1,841 proteins from 3,675 training proteins have 958 domains totally.

Table B. Statistics of reconstructed datasets

	Training dataset	Validation dataset	Test dataset
**# of compounds**	8,751	350	12,810
**# of proteins**	1,841	277	408
**# of positive interactions**	16,194	221	12,996
**# of negative interactions**	16,194	294	10,508

Table C. Selected hyperparameters of implemented DL-CPI

Hyperparameter	Original value	Selected value
**# of hidden layers**	4	2
**# of hidden units per layer**	2000	400
**Sparsity coefficient**	0.0001	0.0001
**Weight penalty coefficient**	0.00001	0.00001
**Optimizer**	Stochastic gradient decent	Adam

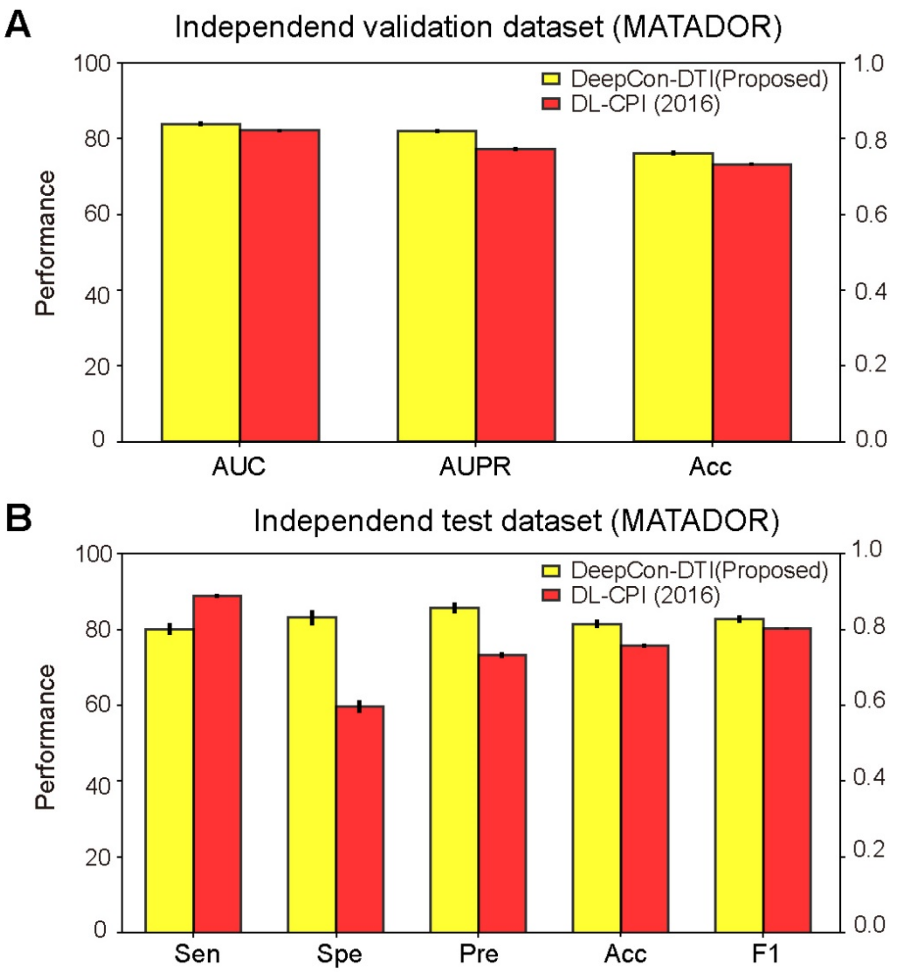

Fig E. Performance comparison between our model and implemented DL-CPI

We optimized hyperparameters of DL-CPI on reconstructed MATADOR dataset. For our model, we used the same hyperparameters as shown in Table A. (**A**) Comparison of optimized performances on MATADOR dataset. (**B**) Comparison of performances of models on the test dataset (PubChem) with the optimized model by validation (MATADOR) dataset.

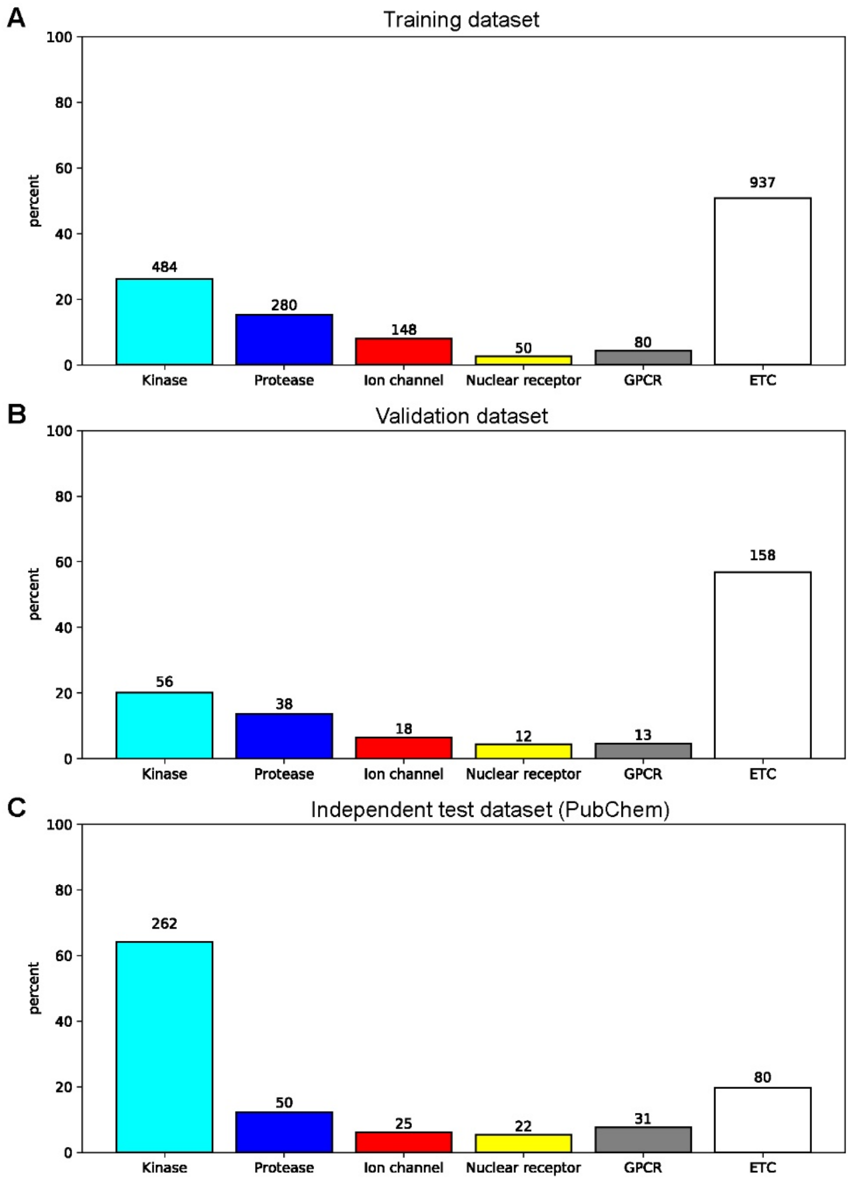

Fig F. Percentages of protein classes in reconstructed datasets.

We examined the percentages of protein classes, consisting of kinase, protease, ion channel, nuclear receptor, GPCR, etc., in each dataset and determined the percentage of each class (**A-C**). Because one protein can have multiple classes, the sum of the percentages can exceed 100%.

Statistics and protein class ratios for datasets

We constructed datasets from various datasets. The training dataset was collected from DrugBank [11], International Union of Basic and Clinical Pharmacology (IUPHAR) [12], and Kyoto Encyclopedia of Genes and Genomes (KEGG) [13] (Table B). The validation dataset was built with MATADOR [14] and negative interactions predicted from *Liu et al*. [15] (Table C). Test datasets were queried from PubChem bioassay [3] and KinaseSARfari [4] (Table D). To show that our datasets (training, validation and test) are not biased to a specific protein class, we summarized the percentage of protein classes, kinases, proteases, ion channels, nuclear receptors, G-protein coupled receptors (GPCRs) and other proteins (etc.) in Fig E. The percentage sum can exceed 100% because one protein can have multiple classes. In addition, we visualized 1,527 proteins used in the training dataset categorized in various protein classes with protein features from the fully connected layer after the global max-pooling of convolution results. Specifically, we visualized 257 GPCRs, 44 nuclear receptors, 304 ion channel receptors, 604 kinases, and 318 protease. For visualization, we conducted t-distributed stochastic neighbor embedding (t-SNE) for dimension reduction and visualization [16]. Surprisingly, although our model is not intended to identify protein classes, it can roughly discriminate protein classes from the intermediate protein layer, as shown in Fig D.

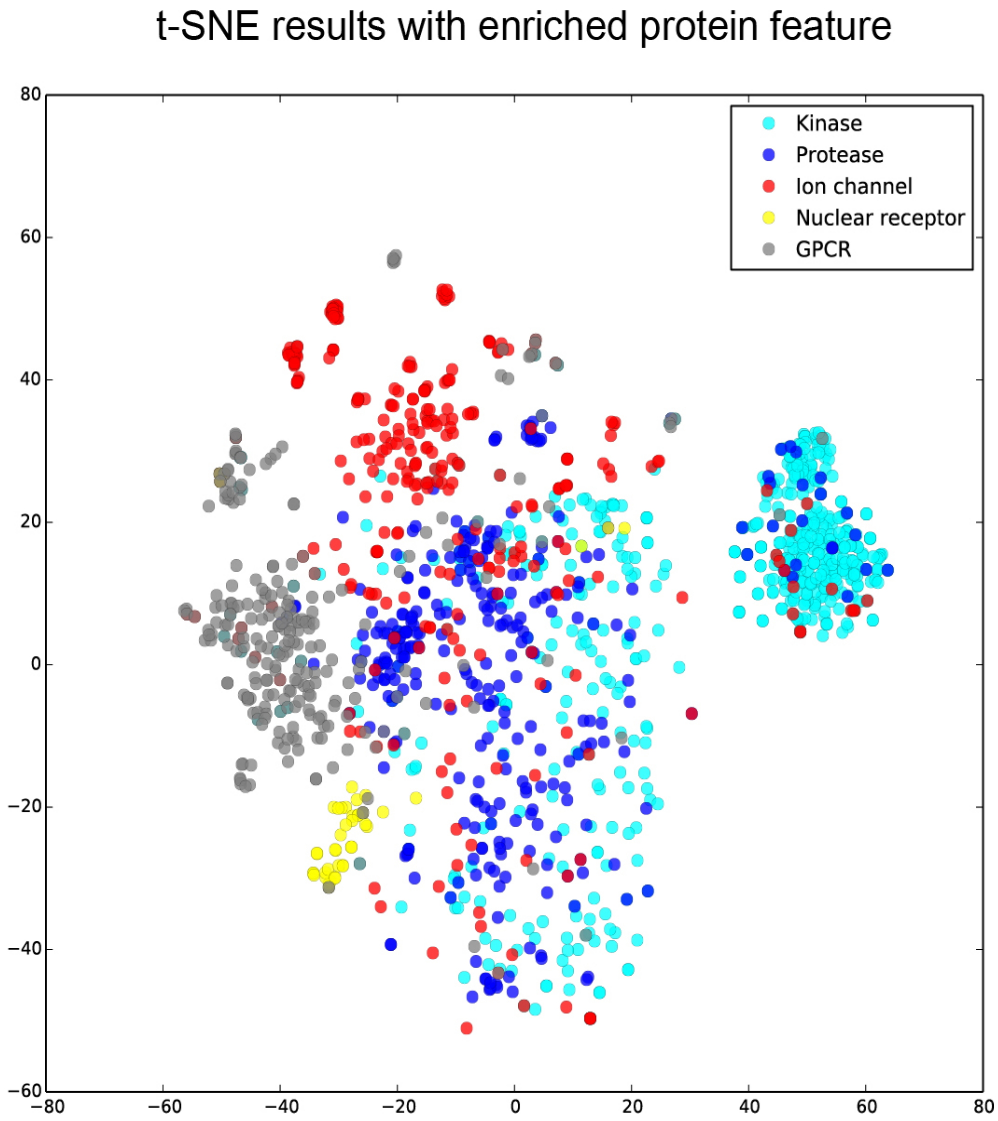

Fig G. t-SNE results with protein features from the fully connected layer beyond the global max-pooling layer of the convolution result.

Each color corresponds to a protein class. The visualization result shows that our model is able to roughly discriminate protein classes by capturing local residue patterns.

Table D. Statistics of the training dataset

	DrugBank	KEGG	IUPHAR	Total
**# of compounds**	4,948	3,696	5,844	11,950
**# of proteins**	2,606	722	1,951	3,675
**# of positive interactions**	12,507	10,968	12,970	32,568
**# of negative interactions**				65,136

Table E. Statistics of the external validation dataset

# of compounds	# of proteins	# of positive interactions	# of negative interactions
499	538	370	507

Table F. Statistics of the independent dataset

	PubChem	PubChem (new compounds)	PubChem (new proteins)
**# of compounds**	21,907	18,043	5,723
**# of proteins**	698	663	119
**# of positive interactions**	18,228	7,740	1,464
**# of negative interactions**	18,228	15,404	6,205

	PubChem (both new)	KinaseSARfari
**# of compounds**	5,600	3,379
**# of proteins**	119	389
**# of positive interactions**	1,464	3,835
**# of negative interactions**	4,980	5,520

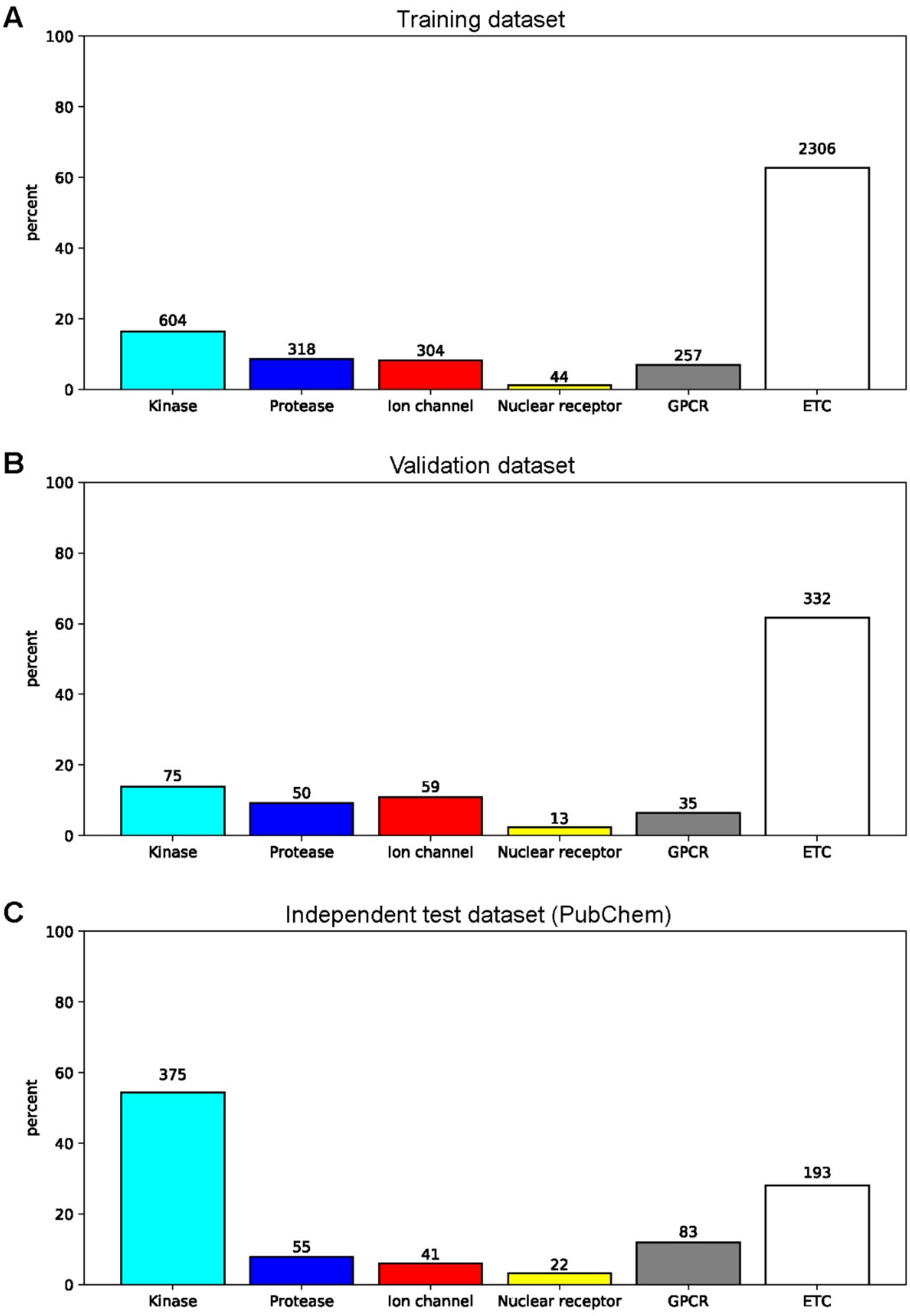

Fig H. Percentages of protein classes in the training, validation, and test datasets.

We examined the percentages of protein classes, consisting of kinase, protease, ion channel,

nuclear receptor, GPCR, etc., in each dataset and determined the percentage of each class (**A-C**). Because one protein can have multiple classes, the sum of the percentages can exceed 100%. The overall ratios of each class in the training and validation sets are similar, but in the test set, kinases represent the largest proportion.

Architecture of the convolutional neural network for protein feature representation

The model starts with an embedding to transform each amino acid into the corresponding embedding vector. The embedding layer is a lookup table of embedding vectors. Embedding vector values are randomly initialized by Xavier initializer (denoted 'glorot normal' in keras), which imposes a normal distribution of weights and variance of output following variance of input [17]. Embedding vectors are trainable, meaning that embedding vector values are also changed to optimize loss during training. From the lookup table, the embedding matrix for the protein sequence is constructed by querying embedding vectors corresponding to amino acids from the embedding layer, as shown in Fig G. For the uniformity of length of the input protein, the null label ($) will be padded to the original sequence until its length reaches the maximum protein length, which is same for all protein inputs. Although our model pads null labels to make sequences have the same length, global max-pooling will filter out those null labels during training and prediction because the process only pools one maximum value from all convolution results from the sequence as depicted in Fig G. As a result, bias is removed from the sequence length variation. In our experiment comparing AUPRs between different max lengths (2,500, 3,000), two models with different lengths do not show significant differences in performance, as shown in Fig A.

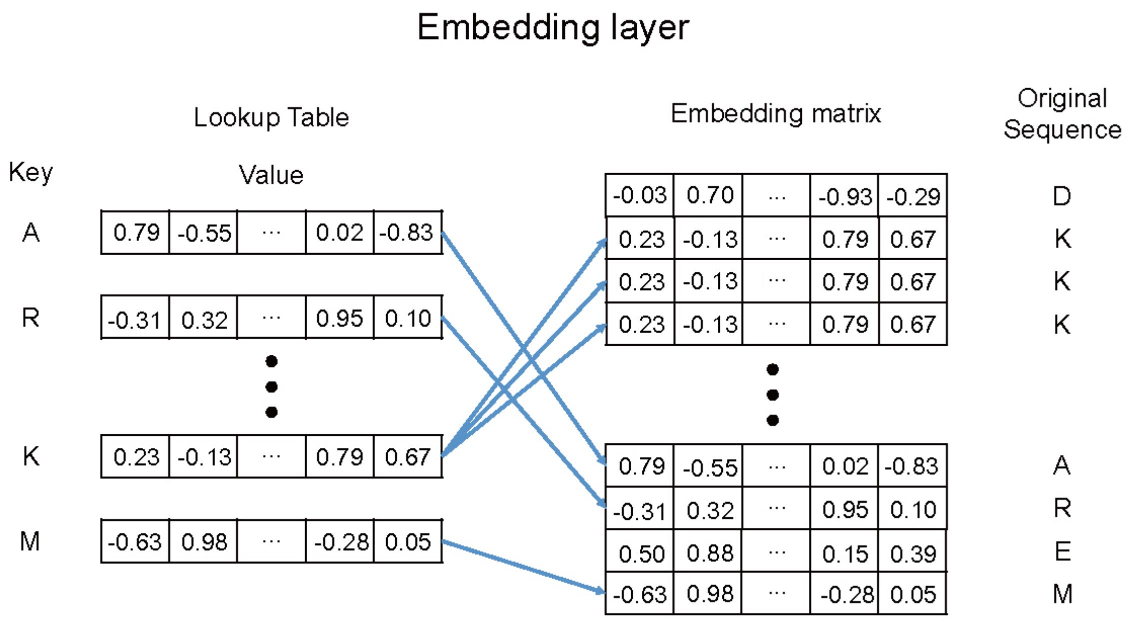

Fig I. Lookup table mechanism in the embedding layer.

The embedding layer is a lookup table of embedding vectors. Embedding vector values are randomly initialized by Xavier initializer (denoted 'glorot normal' in keras), which imposes a normal distribution of weights and variance of output following variance of input. Embedding vectors are trainable, meaning that embedding vector values are also changed to optimize loss during training. From the lookup table, the embedding matrix for the protein sequence is constructed by querying embedding vectors corresponding to amino acids from the embedding layer.

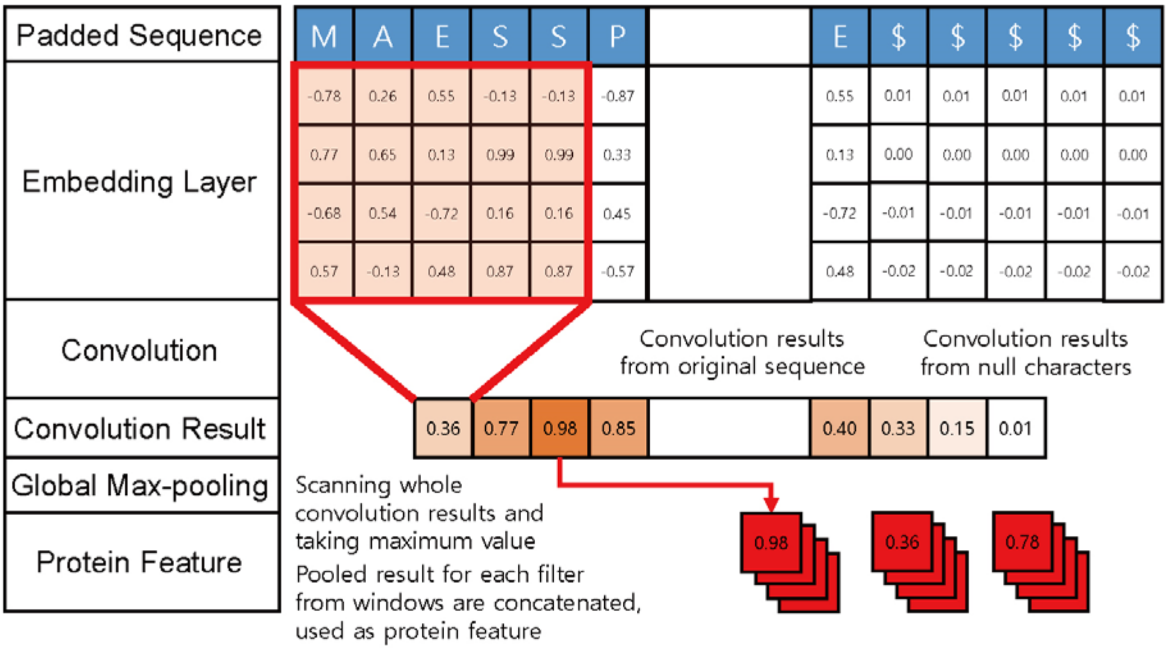

Fig J. Global max-pooling on protein sequence and its embedding layer.

Global max-pooling takes the maximum value from all convolution results on a sequence. When pooling maximum values, convolution results from null characters are filtered out.

References

1. Dubchak I, Muchnik I, Holbrook SR, Kim SH. Prediction of protein folding class using global description of amino acid sequence. Proc Natl Acad Sci U S A. 1995;92(19):8700-4. PubMed PMID: 7568000; PubMed Central PMCID: PMCPMC41034.

2. Smith TF, Waterman MS. Identification of common molecular subsequences. J Mol Biol. 1981;147(1):195-7. PubMed PMID: 7265238.

3. Wang Y, Bryant SH, Cheng T, Wang J, Gindulyte A, Shoemaker BA, et al. PubChem BioAssay: 2017 update. Nucleic Acids Res. 2017;45(D1):D955-D63. doi: 10.1093/nar/gkw1118. PubMed PMID: 27899599; PubMed Central PMCID: PMCPMC5210581.

4. Bento AP, Gaulton A, Hersey A, Bellis LJ, Chambers J, Davies M, et al. The ChEMBL bioactivity database: an update. Nucleic Acids Res. 2014;42(Database issue):D1083-90. doi: 10.1093/nar/gkt1031. PubMed PMID: 24214965; PubMed Central PMCID: PMCPMC3965067.

5. Yamanishi Y, Araki M, Gutteridge A, Honda W, Kanehisa M. Prediction of drug-target interaction networks from the integration of chemical and genomic spaces. Bioinformatics. 2008;24(13):i232-40. doi: 10.1093/bioinformatics/btn162. PubMed PMID: 18586719; PubMed Central PMCID: PMCPMC2718640.

6. Peng W, Chan KCC, You ZH, editors. Large-scale prediction of drug-target interactions from deep representations. 2016 International Joint Conference on Neural Networks (IJCNN); 2016 24-29 July 2016.

7. You ZH, Chan KCC, Hu PW. Predicting Protein-Protein Interactions from Primary Protein Sequences Using a Novel Multi-Scale Local Feature Representation Scheme and the Random Forest. Plos One. 2015;10(5). PubMed PMID: WOS:000354049700088.

8. Tian K, Shao M, Wang Y, Guan J, Zhou S. Boosting compound-protein interaction prediction by deep learning. Methods. 2016;110:64-72. doi: 10.1016/j.ymeth.2016.06.024. PubMed PMID: 27378654.

9. Finn RD, Bateman A, Clements J, Coggill P, Eberhardt RY, Eddy SR, et al. Pfam: the protein families database. Nucleic Acids Res. 2014;42(Database issue):D222-30. doi: 10.1093/nar/gkt1223. PubMed PMID: 24288371; PubMed Central PMCID: PMCPMC3965110.

10. Kingma DP, Ba J. Adam: A Method for Stochastic Optimization. ArXiv e-prints [Internet]. 2014 December 1, 2014; 1412. Available from: http://adsabs.harvard.edu/abs/2014arXiv1412.6980K.

11. Law V, Knox C, Djoumbou Y, Jewison T, Guo AC, Liu Y, et al. DrugBank 4.0: shedding new light on drug metabolism. Nucleic Acids Res. 2014;42(Database issue):D1091-7. doi: 10.1093/nar/gkt1068. PubMed PMID: 24203711; PubMed Central PMCID: PMCPMC3965102.

12. Southan C, Sharman JL, Benson HE, Faccenda E, Pawson AJ, Alexander SP, et al. The IUPHAR/BPS Guide to PHARMACOLOGY in 2016: towards curated quantitative interactions between 1300 protein targets and 6000 ligands. Nucleic Acids Res. 2016;44(D1):D1054-68. doi: 10.1093/nar/gkv1037. PubMed PMID: 26464438; PubMed Central PMCID: PMCPMC4702778.

13. Kanehisa M, Furumichi M, Tanabe M, Sato Y, Morishima K. KEGG: new perspectives on genomes, pathways, diseases and drugs. Nucleic Acids Res. 2017;45(D1):D353-D61. doi: 10.1093/nar/gkw1092. PubMed PMID: 27899662; PubMed Central PMCID: PMCPMC5210567.

14. Gunther S, Kuhn M, Dunkel M, Campillos M, Senger C, Petsalaki E, et al. SuperTarget and Matador: resources for exploring drug-target relationships. Nucleic Acids Res. 2008;36(Database issue):D919-22. doi: 10.1093/nar/gkm862. PubMed PMID: 17942422; PubMed Central PMCID: PMCPMC2238858.

15. Liu H, Sun J, Guan J, Zheng J, Zhou S. Improving compound-protein interaction prediction by building up highly credible negative samples. Bioinformatics. 2015;31(12):i221-9. doi: 10.1093/bioinformatics/btv256. PubMed PMID: 26072486; PubMed Central PMCID: PMCPMC4765858.

16. van der Maaten L, Hinton G. Visualizing Data using t-SNE. J Mach Learn Res. 2008;9:2579-605. PubMed PMID: WOS:000262637600007.

17. Glorot X, Bengio Y. Understanding the difficulty of training deep feedforward neural networks. In: Yee Whye T, Mike T, editors. Proceedings of the Thirteenth International Conference on Artificial Intelligence and Statistics; Proceedings of Machine Learning Research: PMLR; 2010. p. 249--56.
