# Supplementary material for: DeepConv-DTI: Prediction of drug-target interactions via deep learning with convolution on protein sequences
Source: PLoS Comput Biol. 2019 Jun 14;15(6):e1007129. doi: 10.1371/journal.pcbi.1007129 (PMC6594651; doi:10.1371/journal.pcbi.1007129)
Supplement: S1 Fig — (PDF) [file pcbi.1007129.s002.pdf]

A

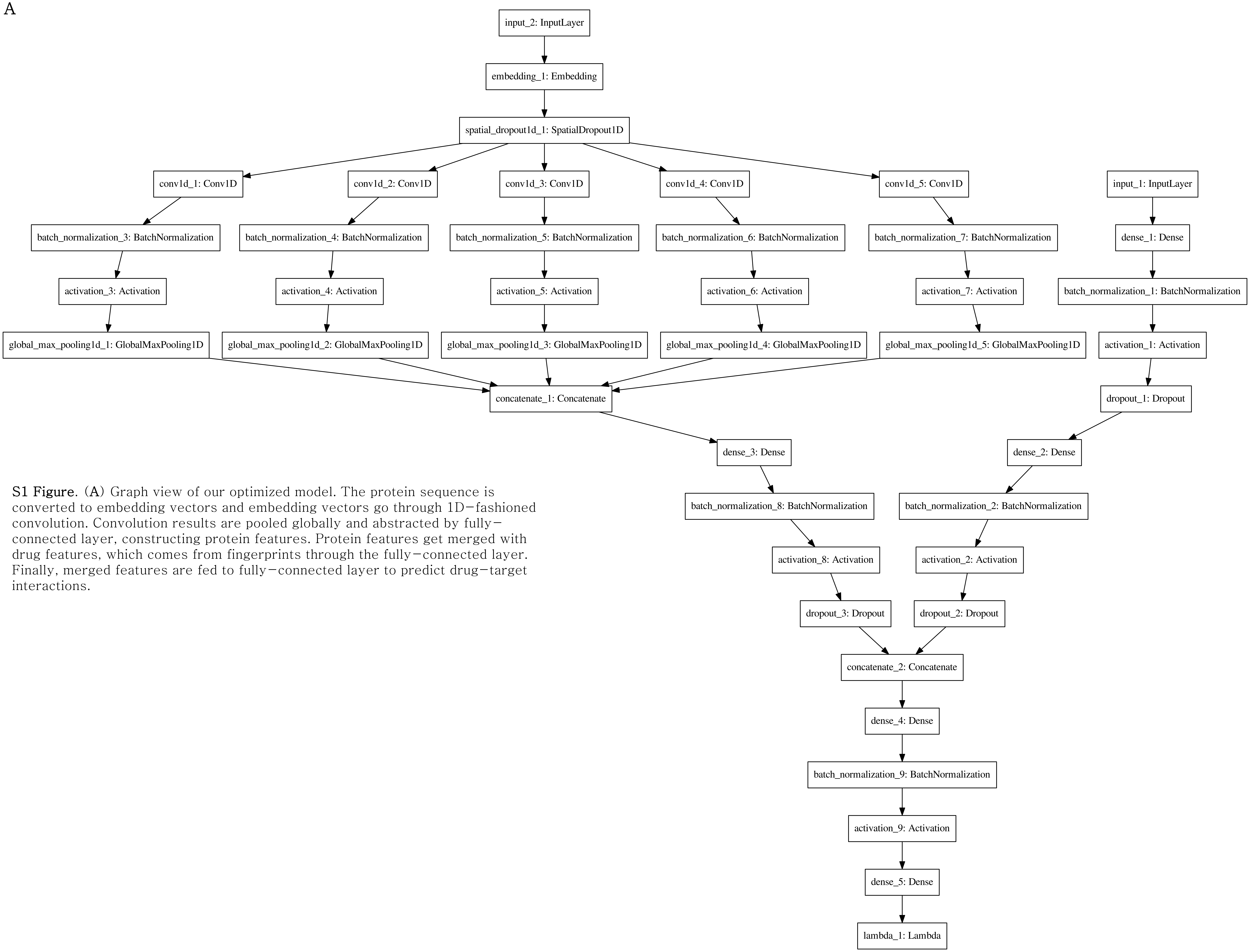

S1 Figure. (A) Graph view of our optimized model. The protein sequence is converted to embedding vectors and embedding vectors go through 1D−fashioned convolution. Convolution results are pooled globally and abstracted by fully−connected layer, constructing protein features. Protein features get merged with drug features, which comes from fingerprints through the fully−connected layer. Finally, merged features are fed to fully−connected layer to predict drug−target interactions.

B

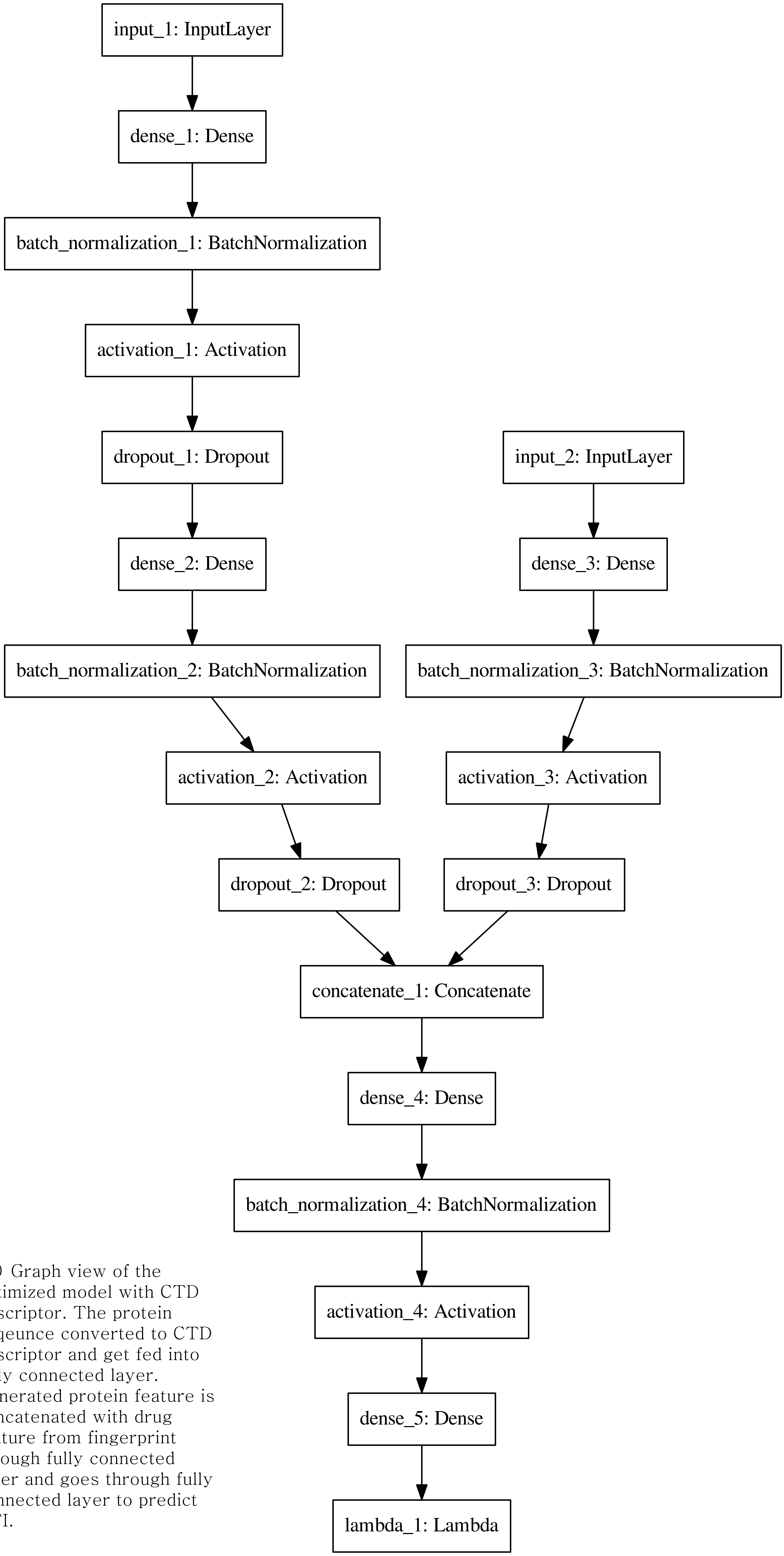

(B) Graph view of the optimized model with CTD descriptor. The protein seqeunce converted to CTD descriptor and get fed into fully connected layer. Generated protein feature is concatenated with drug feature from fingerprint through fully connected layer and goes through fully connected layer to predict DTI.

C

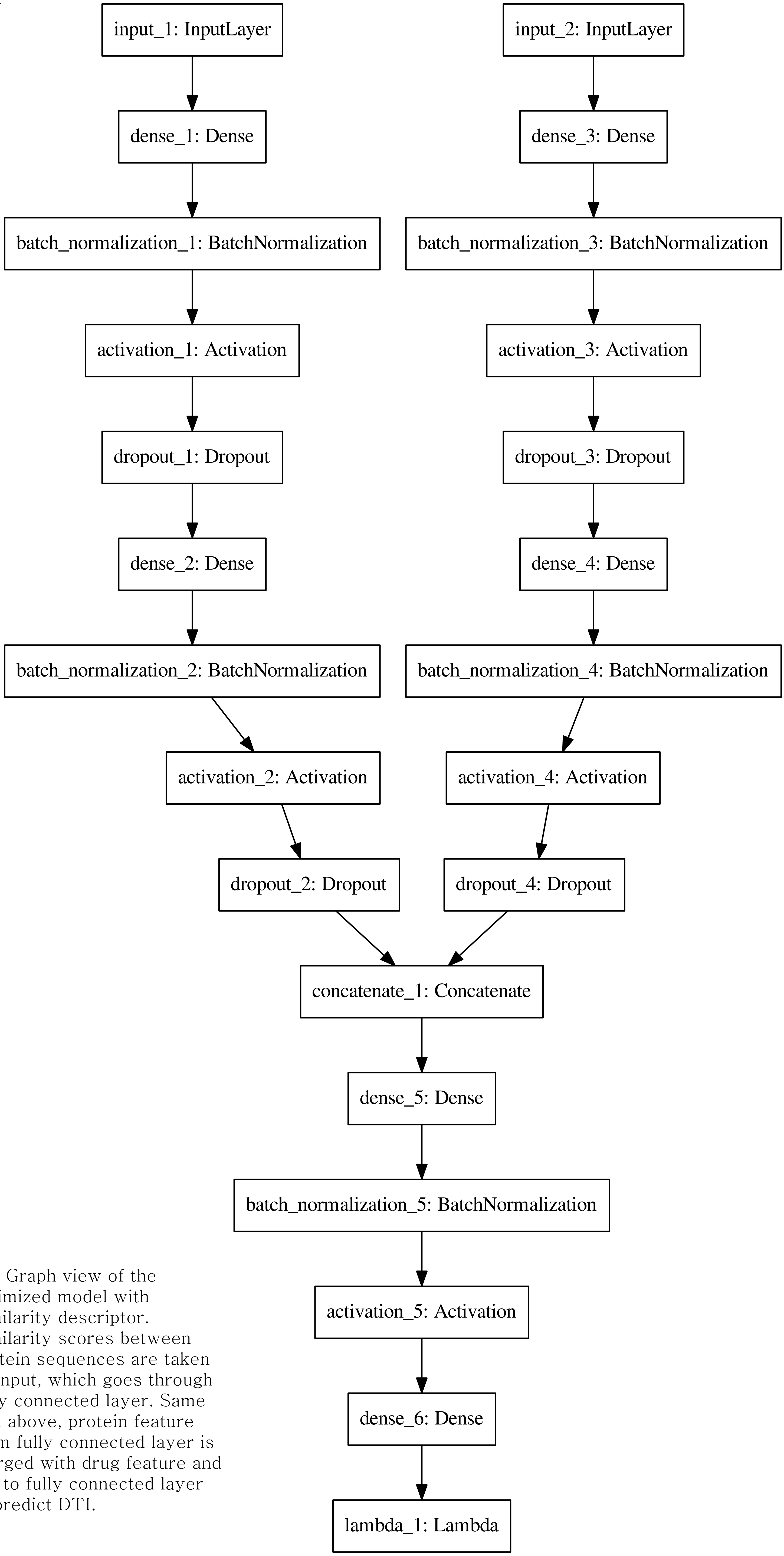

(C) Graph view of the optimized model with Similarity descriptor. Similarity scores between protein sequences are taken as input, which goes through fully connected layer. Same wth above, protein feature from fully connected layer is merged with drug feature and fed to fully connected layer to predict DTI.
